# Three Major Causes of Metabolic Retinal Degenerations and Three Ways to Avoid Them

**DOI:** 10.3390/ijms24108728

**Published:** 2023-05-13

**Authors:** Andrea Kovács-Valasek, Tibor Rák, Etelka Pöstyéni, Adrienne Csutak, Robert Gábriel

**Affiliations:** 1 Department of Experimental Zoology and Neurobiology, University of Pécs, Ifjúság útja 6, 7624 Pécs, Hungary; 2 Department of Ophthalmology, Medical School, University of Pécs, Szigeti út 12, 7624 Pécs, Hungary; 3 János Szentágothai Research Centre, University of Pécs, Ifjúság útja 20, 7624 Pécs, Hungary

**Keywords:** retinal neurons, retinal vasculature, Müller glia, retinal pigment epithelium, anti-apoptotic pathways, plant extracts, protective factors, retinoprotection, visual functions

## Abstract

An imbalance of homeostasis in the retina leads to neuron loss and this eventually results in a deterioration of vision. If the stress threshold is exceeded, different protective/survival mechanisms are activated. Numerous key molecular actors contribute to prevalent metabolically induced retinal diseases—the three major challenges are age-related alterations, diabetic retinopathy and glaucoma. These diseases have complex dysregulation of glucose-, lipid-, amino acid or purine metabolism. In this review, we summarize current knowledge on possible ways of preventing or circumventing retinal degeneration by available methods. We intend to provide a unified background, common prevention and treatment rationale for these disorders and identify the mechanisms through which these actions protect the retina. We suggest a role for herbal medicines, internal neuroprotective substances and synthetic drugs targeting four processes: parainflammation and/or glial cell activation, ischemia and related reactive oxygen species and vascular endothelial growth factor accumulation, apoptosis and/or autophagy of nerve cells and an elevation of ocular perfusion pressure and/or intraocular pressure. We conclude that in order to achieve substantial preventive or therapeutic effects, at least two of the mentioned pathways should be targeted synergistically. A repositioning of some drugs is considered to use them for the cure of the other related conditions.

## 1. Introduction

The visual world is the most important environmental information source for humans. None of the other sensory signals reaches the brain in such variety and none is processed by as many cortical areas as the visual cues [1,2]. The first steps of visual processing however do not happen in the brain; these are performed by a thin sheath of neural tissue at the back of the eye, called the retina. After phototransduction by photoreceptors (PRs), light information is translated into neural signals. Visual signals to the brain are solely of retinal origin. They are generated by the retinal ganglion cells (RGC) in the form of spike trains [3]. Therefore, any damage to the retinal tissue immediately results in vision loss and, in the worst case, causes total blindness.

Sight-threatening neurodegenerative diseases of the retina fall into two broad categories; one group is caused by genetic deficits, e.g., retinitis pigmentosa and microphthalmia [4]. Other retinodegenerative disorders such as age-related degenerations (age-related macular degeneration—AMD), diabetic retinopathy (DR) or glaucoma are thought to be consequences of pathological metabolic processes [5]. These three conditions contribute strongly to blindness causes worldwide. Exposure to extremely strong light or UV radiation, changes in metabolic parameters or hormone secretion or eventually chronic high blood pressure (resulting in elevated intraocular pressure (IOP)) all may cause tissue metabolic changes. These processes induce elevated extracellular glutamate levels, provoking excitotoxic insults [6]. Besides apoptosis induced by the excitotoxic insults, other means of retinal deteriorations have also been studied in many laboratories. These include reactive oxygen species (ROS)-induced alterations [7,8,9], inflammatory/parainflammatory processes [10,11,12], vascular endothelial growth factor (VEGF)-mediated changes in retinal blood vessels [13] and a regulation of autophagic/necrotic events [14].

**Figure 1:**
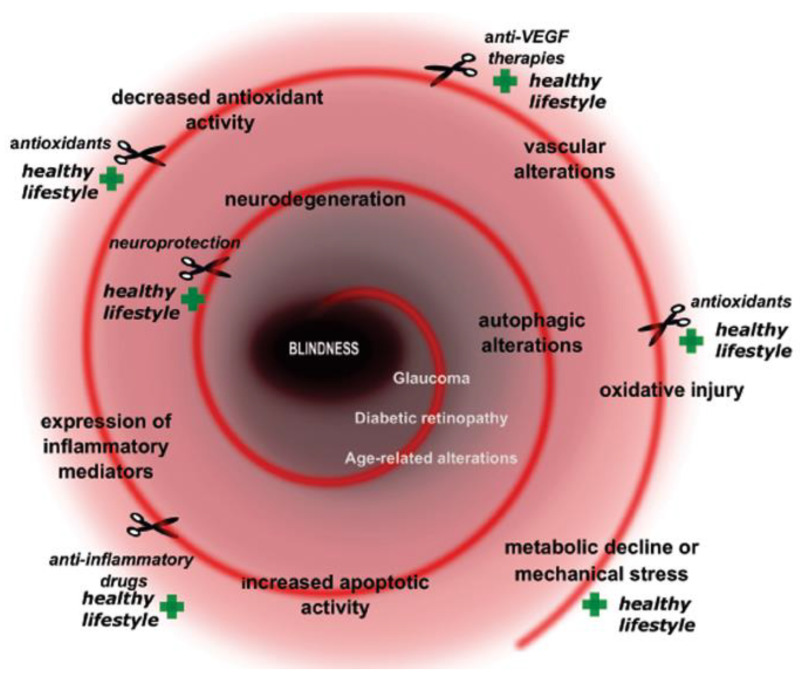
Key elements and prevention/cure points in metabolically induced retinal diseases. Plus signs show prevention points, while scissor signs demonstrate cure or preventation possibilities.

Neuronal viability of the retina is based on several fundamental homeostatic processes and discrete signaling pathways. Its neuronal complexity, high metabolic demand and limited regenerative capacity make it extremely vulnerable against a variety of intracellular and extracellular stress factors. If the stress threshold is exceeded, different protective/survival mechanisms are activated. Metabolic-related stresses could manifest at different levels and with diverse, severe consequences in the retina. Numerous key molecular actors contribute to the evolution of growingly prevalent metabolically induced retinal diseases. These diseases have a complex dysregulation of glucose-, lipid-, amino acid or purine metabolism in the molecular background of the pathology [15]. Several altered metabolic stress-related cellular pathways are induced such as oxidative stress [16], defects in autophagy and immune cascade, mitochondrial dysfunction, endoplasmatic stress, alterations in apoptotic pathways etc. Moreover, neuroinflammation, glial activation and glutamate excitotoxicity also could be main players in the development of the pathogenesis (Figure 1).

Under physiological conditions, homeostatic parameters are in perfect balance. During the development of diseases, harmful or even toxic substances (see later) may be generated, which force the tissue to produce protective compounds. If the elements of the reina tissue (Müller cells, retinal vascular, endothel, microglia and even the retina nervous cells) react properly, the survival chances of retinal neurons increase [7,15].

The prevalence of vision loss will double by 2050 [17], from which the “big three”, AMD, DR and glaucoma, will have a substantial share; therefore, solutions for prevention that are widely available for patients are urgent. As indicated in the title above, we aimed at finding the common elements of the signalling pathways which cause age-related changes (most particularly AMD) and those disorders that may lead to blindness due to the deterioration of the retina: diabetes and glaucoma.

## 2. Aims

In this review, therefore, we summarise current knowledge on possible ways of preventing or circumventing retinal degeneration by available methods. We intend to provide a common treatment rationale for these disorders and to collect the possible mechanisms through which these actions protect the retina. To specify further, we shall consider (1) the possible role of natural products and complementary approaches to prevent the above disorders; (2) to review the endogenous substances in the service of retinal integrity and ways to use them for prevention and cure; and (3) to list synthetic drugs and other chemical means to protect the retinal circuitry. The successful efforts to use non-invasive or minimally invasive combination treatments (4) will also be considered. Finally, we shall summarise the common mechanisms (5) of the three disorders and point at the critical targets which can be attacked successfully in future therapeutic efforts.

## 3. Retinal Health: The Major Metabolic Threats to It and Ways to Avoid Damage

Since the 1900s, psychosomatic and mental factors have also been considered and confirmed to play a role in vision loss due to cataract and retinal disorders such as AMD, DR and glaucoma. These three disorders are responsible for the majority of blindness of retinal origin, and in the later parts of this paper, we shall call them the “big three”. Psychological and neurological aspects can be explained by the development of the fetal eye during embryogenesis: embryologically, the lens, corneal epithelium, neuroretina, brain and skin (eyelid as well) are ectodermal organs. Therefore, it is not surprising that almost all neurodevelopmental and psychiatric disorders affect the skin and eyes, becoming more multisystemic, explained by the same genetic factors (*PAX6, SIX3, SEMA3A* etc.) [18,19,20,21,22]. Mentioning a few relevant examples, neurofibromatosis is an inherited neuro-oculo-cutaneous syndrome affecting all parts of the ectoderm (nervous system, skin and the eye) [19]. Moreover, a higher incidence of mental illness and disabilities with behavioural abnormalities is also related to ocular structural anomalies [23]. One of the most surprising phenomena was the association between atopic dermatitis and ocular complications, including (subcapsular) cataract with an additional consequent retinal detachment. In the case of ocular symptoms, patients typically experience anxiety and a fear of becoming blind, which creates a psychological burden [24]. It is also known that a psychosocially stressful event contributes to an acute myopic shift in refraction in young adults, which suggests that acute mental stress factors may play a role in refractive errors [25]. A higher prevalence of depression was also found in AMD patients, and schizophrenic patients have significantly reduced retinal thickness and retinal blood flow [24,26]. Stress may play a greater role in younger patients with glaucoma, which is both an ocular and brain neurodegenerative disorder characterised by a progressive damage of the optic nerve. The glucocorticosteroid overexposure of the brain can become toxic to neurons and can even be toxic to the retinal tissues. Because of the feedback loops, glucocorticoid increase progressively damages the tissue, as a vicious cycle. Psychosocial stress can also activate inflammatory responses by neural activation of signaling pathways, resulting in an increased nuclear factor κB (NFκB) production. In glaucomatous and healthy eyes, mental stress is associated with IOP elevation and vascular dysregulation which are caused by the glucocorticoids, pro-inflammatory cytokines, and endothelin. Elevated levels of tumor necrosis factor (TNF)-α and interleukin (IL)-6 are found in the aqueous humour too [24]. These factors may all contribute to the loss of metabolic control. If the metabolic shift is slow, aging gains ground; if some of the changes are prominent, retinal diseases can be diagnosed. The most prominent ones worldwide are AMD, DR and glaucoma.

## 4. Aging

Over the past 50 years, rapid changes can be observed in the demographics of populations around the world due to increasing longevity. According to ‘The 2017 World Health Organization World report’, the number of people aged 60 years or older will be 1 billion in 2019. This number is expected to pass to 1.4 billion by 2030 and 2.1 billion by 2050, which at that time will represent close to 22% of the global population. Many of the consequences of population aging are common across human societies all around the world. Therefore, the prevalence of people with major eye conditions that cause vision impairment escalates sharply. There are currently at least 2.2 billion people who have a near or distance vision impairment including AMD (196 million), DR (146 million) and glaucoma (76 million) patients [27]. The incidence of early and late AMD was 4.1% and 2.3%, respectively, with the incidence of early and late AMD being highest in Caucasians (5.3% and 4.1%), intermediate in Asians (4.5% and 2.2%) and Hispanics (3.3% and 0.8%), and lowest in African Americans (1.6% and 0.4%). Adjusting for age and sex, African Americans had a 70% lower risk of developing early AMD than Caucasians, and this decreased only slightly to 67% lower risk after multivariable adjustments [28]. In 2009, the SEARCH Study for Diabetes in Youth determined a prevalence per 1000 for youths aged 0–19 years with type 1 diabetes from different ethnic groups in the United States. Type 1 diabetes remains a white-dominated disease, with rates of 2.00 in non-Hispanic white patients, 1.31 for African Americans, 0.99 for Hispanics, 0.94 for Native Americans and 0.52 for Asians and Pacific Islanders. For type 2 Diabetes, similar ratios were found [29]. The global prevalence of glaucoma was roughly 3.5% for people aged 40–80 years. The prevalence of primary open-angle glaucoma (POAG) was highest in Africa (4.2%), and primary angle-closure glaucoma was most prevalent in Asia (1.1%) [30].

### 4.1. Progression of Retinal Aging–Common Features in Model Species and Humans

The changes in visual function induced by aging have been commonly observed in several species such as mice, rats, sheep, degus, monkeys and humans [31,32,33,34,35,36,37,38,39,40,41,42,43,44,45,46,47,48,49,50,51]. Each component of the eye and, within it, the retina contribute differently to the overall decline in visual function with aging. Remarkable morphological changes occur that are primarily manifested by thinner retinal layers due to cell loss and/or synaptic connection alterations. Some studies have reported age-related ganglion cell loss [31,32,49,52,53,54] while others have not [34,39,44,55]. Nuclear densities declined more intensively in the outer nuclear layer than in inner nuclear layers [56]. Rod bipolar cell dendrites and horizontal cells sprouted into the PR layer most abundantly in the peripheral retina [35,57,58]. The generation of these aberrant processes is presumably driven by the PR death or loss of PR function. It is well established that PR density decreases with age: cones were more resistant than rods to destruction by age and ambient light [32,36,56,59]. Some studies demonstrate that the survival of cones depends on the presence of rods, even if these rods are not functional, as rods secrete cone survival factors [60,61,62,63]. However, at very advanced ages, a significant loss of central cones was also described [33,57,64]. The decline in rod density is supposed to be due to the decline of the neurons which are connected to them (such as the rod bipolar cells in the outer plexiform layer) [36]. Retinal pigment epithelium (RPE) neovascularization from retinal vessels appears also to be characteristic of most animal models undergoing PR cell degeneration [65]. In humans, age-related macular degeneration (AMD) is a complex eye disease. The overall prevalence of age-related macular degeneration is estimated to increase 7-fold, from 4.2% in those aged 45–49 years to 27.2% in those aged 80–85 years [66]. Based on the appearance of specific symptoms, we can speak about the more common non-neovascular (dry, nonexudative) AMD or the less prevalent neovascular (wet, exudative) types [67]. However, it is difficult to find the border between the changes caused by aging and the start of the pathology; several classifications or grading systems are known. The guidance of the National Institute for Health and Care Excellence (NICE) recommends distinguishing three states, depending on the presence, size and approximate number of drusen, the degree of neovascularization and pigmentary abnormalities. These are namely early, intermediate and advanced [68]. In 2008, Takahashi et al. described new classifications and diagnostic criteria for AMD therapy. According their suggestion, the prodominal stage can be divided into soft drusen and retinal pigment epithelial abnormalities, and AMD is classified into exudative, atrophic macular degeneration [69]. The Clinical Age-Related Maculopathy Staging System (CARMDS) classifies AMD into 1 of 5 mutually exclusive stages [70], while Sandberg’s system suggests four grades. In addition, the Age-Related Eye Disease Study (AREDS) recommends a nine- and a four-step scale for classifying the stage of the disease [71].

### 4.2. Cellular and Subcellular Changes

In the RPE, one other major marker of aging is the abnormal accumulation of lipofuscin that originates from the incomplete phagocytosis of the PR outer segments. Accumulation of the undegradable end product generates superoxide ions, singlet oxygen, hydrogen peroxide and lipid peroxides [72]. The lipofuscin fluorophore A2E mediates the apoptosis of RPE in a wavelength-dependent manner. Blue light (400–520 nm) generates more superoxide anions than red light (660–730 nm) or full white light [73]. In addition to the intracellular deposit of lipofuscin, an extracellular deposit also appears in the retina with age that is known as drusen. This debris is localized between the RPE basal lamina and the inner collagenous layer of the Bruch’s membrane. Drusen mainly contains proteins such as the serum amyloid P component, apolipoprotein E, vitronectin, immunoglobulin light chains, factor X, complement proteins and Alzheimer’s Ab-peptide [74]. Permeability of Bruch’s membrane and vascular basal membrane also decreases with age due to the accumulation of esterified cholesterol [75].

These morphological changes correlate strongly with functional disparities. Electroretinogram (ERG) analysis in the retina revealed the reduced amplitudes of rod- and cone-mediated responses in the elderly [35,76,77,78,79]. These studies also concluded that functional discrepancies measured by ERG could be due to a reduction of the number of PRs but even more, the mislocalization of PR synaptic terminals that caused a decline in PR transduction efficiency [79]. The amplitude of multifocal oscillatory potentials, which are thought to reflect inner retinal function and the rod–cone interaction, decreases linearly, whereas their latency increases with age [59,80]. Other investigations suggest an altered inflammatory response with age. Several inhibitory molecules keep retinal microglia and the complement system under tight control in normal physiological conditions.

### 4.3. Molecular Considerations

Just like cells in other organ systems, retinal cells suffer from accumulative oxidative load and metabolic stress in the aging process. Increased oxidative stress and the accumulation of damaged molecules lead to the dysfunction of various metabolic and signaling pathways which, in the retina, ultimately result in pathologies such as glaucoma or age-related macular degeneration, as well as the neuropathic complications of diabetes in the eye. The ROS particles originate mainly from mitochondria as part of the production of ATP by the classical glucose oxidation pathway, but mitochondria also regulate the intracellular pH, calcium concentration and contribute to apoptotic signaling pathways [81]. mtDNA mutations often associate with cytochrome c oxidase-deficient cones and ever more accumulate in PRs with age, mostly in the foveal region [82]. The partial loss of proteasomal function and expression are also typical phenomena in the retinae of elderly rats [80,83]. Proteins originated from drusen were also rich in post-translational oxidative modifications, such as carboxyethylpyrrole protein and advanced glycosylation end products (AGEs) [84]. Other investigations suggest an altered inflammatory response with age through the activation of the receptor of AGE [80]. Elevated levels of AGEs have also been found in retinal blood vessels, serum, and vitreous of diabetic patients. Several inhibitory molecules keep retinal microglia (certain interleukins) and the complement system under control in normal physiological conditions [85].

Besides the complex processes which occur during aging, aformentioned age-related structural and functional alterations show similarities with the initial phase of pathologies in many cases. To recognize the disparities and border between normal aging and the pathological state can be complicated but provides valuable information for the prevention or compensation of the processes of disease development. Two such major disorders are DR and glaucoma (see Section 5 and Section 6).

### 4.4. Preventive and Therapeutic Measures during Aging

The best characterized age-related retinal complication is AMD. A strong evidence-based empirically developed antioxidant formulation was introduced in the AREDS (Age-Related Eye Disease Studies). The effects were analysed in a blinded, randomized and controlled study on several thousand patients. The original AREDS formulation contained β-carotene, vitamin C, vitamin E and zinc, among other components. Further nutrients and nutraceuticals with antioxidant, anti-inflammatory and anti-apoptotic properties have been extensively studied to employ them for neuroprotection [86]. Immunostimulatory effects of some herbal compounds have been documented which include increased levels of cytokines (IL-2 and TNFα), resulting in retinal microglia activation. Pharmacophytones also interact with anti-apoptotic pathways and decrease proapoptotic pathways (e.g., NFκB, caspase activity), thereby preventing apoptosis and autophagy [87]. Herbal formulas and plant extracts may also exhibit anti-angiogenic effects by inhibiting VEGF signal transduction [88]. Thus, herbal extracts initiate various mechanisms to preserve the retina from neurodegeneration [87]. Table S1 in the Supplementary Materials collects herbs and active agents applied on humans and animals in connection with the therapy of retinal degenerations, such as AMD, DR and glaucoma [18,86,87,88,89,90,91,92,93,94,95,96,97,98,99,100,101,102,103,104,105,106,107,108,109,110,111,112,113,114,115,116,117,118,119,120,121,122,123,124,125,126,127,128,129,130,131,132,133].

Epidemiological evidence has shown that patients with lower concentrations of macular pigment optical density measurements are at a higher risk of developing AMD [94]. Participants with the highest self-reported dietary intake of a mixture of vitamin A, vitamin C and vitamin E had a larger effect on the reduction of AMD risk than the individual vitamins [134].

Common pathophysiological pathways of ocular pathologies can be established due to metabolic stress that causes an elevated glutamate realisation and an insufficient supply of nutrients to the respective target structure, mainly on ganglion cells and a specific type of amacrine cells which contain ionotropic glutamate (N-methyl-D-aspartate, NMDA) receptors. Several potentially neuroprotective drugs have already been administered to reduce or prevent the death of retinal neurons. Table S2 in the Supplementary Materials [135,136,137,138,139,140,141,142,143,144,145,146,147,148,149,150] collects synthetic drugs applied on humans and animals in connection with the therapy of retinal degenerations, such as AMD, DR and glaucoma. The mode of action is mainly to block NMDA receptors or to interrupt the influx of calcium and sodium into cells to prevent the generation of free radicals linked to the formation of AGEs and/or advanced lipoxidation end products, as well as to avoid defects in the mitochondrial respiratory chain [151]. To evaluate current and new drugs that are neuroprotective, the following criteria must be met: there is a specific target in the retina/optic nerve; the drug must reach the retina at levels sufficient to activate its target; it must have a mechanism of action that enhances a neuron’s resistance to stress or suppresses toxic insults; and there should also be demonstrated activity in human clinical trials.

Recently, a transgenic mouse model has been developed where somatostatin (SST)—containing amacrine cells—have been tagged with the red td-tomato fluorescent marker. This way, the fate of these cells could be followed during the aging process. SST is an important marker of retinal health since it is downregulated in the diabetic eye, and SST-containing eye-drop treatment prevented ERG abnormalities, glial activation, apoptosis and a misbalance between pro-apoptotic and survival signaling in rats with DR [152]. If these animals received chronic pituitary adenylate cyclase-activating polypeptide (PACAP) injections every three months, the loss of SST cells from the aging retina could be reversed [153]. PACAP was known for a long time to inhibit the development of DR through increasing anti- and decreasing pro-apoptotic factors in different retinal and other injuries [154]. This mechanism involves also the upregulation of PAC1 receptors on dopaminergic amacrine cells of the retina [155]. Since the 27 amino acid forms of PACAP could be applied successfully in eye drops [156,157], the possibility of a convenient non-invasive preventive treatment option arises.

## 5. Diabetic Retinopathy

More than 536 million people were diagnosed with diabetes in 2021, and this will reach 783 million in 2045 [158]. Globally, an increasing number of patients with diabetes also predicts the rising prevalence of DR. DR is the leading cause of preventable vision-loss in the working population and affects one-third of diabetic patients [159], and 90% of patients will have DR within 25 years of diagnosis [160]. It is a complex, multifactorial neurovascular disease. Early and advanced stages can be distinguished during the disease development. In the advanced phase, the structural lesion is significant, and the visual acuity is also affected.

### 5.1. Risk Factors and Disease Progression

At the beginning of the pathomechanisms, hyperglycemia promotes microvascular degeneration and blood-retina-barrier (BRB) breakdown. Apoptosis of pericytes and endothelial cells, capillary occlusion and increased vascular permeability are also present at the early non-proliferative phases of DR. The microcirculatory pathology generates a hypoxic/ischemic environment in the retina. Due to the insufficient oxygen supply and hyperglycaemia, ROS production increases, and it accelerates several intracellular alterations such as oxidative stress, endoplasmic reticulum stress and mitochondrial dysfunction. Moreover, neuron-glia interaction changes and leads to a global neurovascular unit (NVU) impairment [161]. NVU consists of glial cells, neurons and a vascular network of the retina, and its damage causes the disruption of multiple retinal cell types. Oxidative stress aggravates the NVU impairment, and the expression of inflammatory mediators and VEGF level increases. The formation of neovascularization and vascular leakage means the beginning of the proliferative phasis. The BRB breakdown causes diabetic macular edema (DME) which is one of the leading causes of vision loss in DR besides neovascularization [161,162]. Vascular abnormalities (vascular leakage, neovascularization, venous dilatation, capillary hyperpermeability, reduced reperfusion etc.), an accumulation of subretinal or intraretinal fluid, decreased oxygen and nutrients supply and macula thickening are the main constituents of the DME pathomechanism [163,164]. Its classification is based on the angiographic pattern (focal or diffuse) [165,166] or on optical coherence tomography images (serous retinal detachment, diffuse thickening, cystoid macular edema) [167,168]. Their treatments are based on intravitreall pharmacological interventions and device-assisted operations (laser photocoagulation, victreoctomy) (see later in Section 7.1, ‘Device-assisted minimally invasive therapy plus drug application approach’ paragraph).

### 5.2. Cellular Stress Response: Oxidative Stress and Autophagy in DR

Chronic hyperglycaemia induces the alterations of the main molecular pathway networks such as polyol-, AGE-, hexosamine-, ROS-, PKC pathways. These are closely related to the dysregulation of autophagy, mitochondrial dysfunction, oxidative stress, glial activation and inflammation in the progression of DR [169,170]. These biochemical and cellular causative elements are responsible for neuronal alterations (neurodegeneration, neuronal apoptosis, altered neuronal communication, gliosis) and also for vascular degradation (BRB disruption, ischemia, neovascularization) [171]. Their causative role in the development of DR highlights the importance of research, which reveals the mechanisms pointing toward promising therapeutic targets.

Prolonged hyperglicemia, advanced glycation and hypoxia promotes the accumulation of ROS and causes endoplasmatic reticulum stress which are responsible for autophagy flux impairment and apoptosis [172,173]. Increased ROS production leads to an imbalance in redox homeostasis which results in neuronal cell loss, vascular abnormalities and the occurrence of advanced DR [16,174].

Autophagy belongs to the essential cellular homeostatic pathways which mediate degradation and recycling of intracellular compartments via lysosomal degradation. It has a meaningful role in the intracellular quality control by removing damaged or toxic cellular constituents and promotes the production of required metabolites. As a response to stress, dysregulation of autophagy was described in several retinal diseases and became an important therapeutic target in treatment strategies [171]. Triggered autophagy contributes to neovascularization, cell death, vascular damage and BRB breakdown [175,176]. The modulation of autophagy has a complex role in the pathomechanism of DR. On the one hand, it reduces apoptosis and neurovascularization but on the other, its pro-apoptotic effect has also been described. This dual phenomenon depends on the strength of cellular stress: it is pro-survival and anti-apoptotic in the early stage of DR but meanwhile, when the cellular stress is stronger, it could also provoke cell death in the advanced stage [177,178]. Lopes and his colleagues have described autophagy dysregulation, a higher VEGF expression and apoptotic rate in Müller cells under high glucose conditions; moreover, rapamicin helped to avoid those [179]. Autophagy has increased in RPE cell culture under high glucose conditions, and the inhibition of autophagy caused increased ROS levels [180]. Increased autophagy has also been described in induced diabetic mice retinas [181,182] and rats [183] and the blockade of autophagy has resulted in neuron protection.

### 5.3. Immune Response: Neuroinflammation and Glial Cell Activation in DR

The retina is immune privileged and usually, activation of retinal glial cells is the first reaction to harmful stimuli. Microglial cells are the main reactive immune cells and important homeostasis keepers in the retina. This type of cell monitors the retinal environment and responds effectively to different types of challenges by changing their activation status. They change their morphology, immunoreactivity and migration based on complex microglial–neuronal contact [184,185,186,187]. At the beginning, they try to maintain tissue integrity but as a consequence of prolonged stress, they become overly activated and express several inflammatory markers (chemokines, cytokines, cytotoxins). Hyperglycaemia, hypertension, oxidative stress, apoptosis, AGE and advanced lipoxidation end products production are together responsible for the induction of the inflammatory signaling in DR [188,189]. Inflammation is a nonspecific response of the immune system to harmful stimuli and one of the prominent factors in DR. This immune activation is responsible for structural and functional alterations in DR, which was identified as a chronic low-grade inflammatory disease of the retina [190,191,192]. Several inflammatory mediators, such as leukoctyes, cytokines [193], adhesion molecules [194,195], growth factors [196] and chemokines [197,198] contribute to the formation of the inflammatory milieu [199]. In a rat DR model, microglial cell status and morphology changed with the progression of diabetes [200,201]. Müller cells also express cytokines (IL-1β—Il-6, IL-8, TNFα), growth factors [202] and several other related factors (such as inducible nitric oxide synthase, prostaglandin E and glial fibrillary acidic protein (GFAP)) under high glucose conditions [190,203,204,205,206,207].

### 5.4. Therapeutic Approaches

Non-synthetic compounds of natural origin are a popular approach in treating diseases. In the PubMed database alone, there are close to one million entries for the isolation and use of such substances from antimicrobial purposes to cancer research. For DR alone, more than 200 extracts were tried with more or less success.

#### 5.4.1. Flavonoids, Polyphenols and Other Natural Antioxidants (Table S1)

Flavonoids and polyphenols function as terminators of free radical chains and chelators of redox-active metal ions catalyzing lipid peroxidation. Baicalein (5,6,7-trihydroxy-2-phenyl-4H-1-banzopyran-4-one) is a flavonoid originally isolated from the roots of *Scutellaria baicalensis*. [208]. With its anti-inflammatory properties, this compound blocks high glucose- induced microglial and astroglial activation and VEGF overexpression, thereby preventing the secretion of inflammatory and/or cytotoxic factors, consequently protecting neurons and vasculature from damage in DR [190]. Eriodictyol is a strong antioxidative flavonoid extracted from *Eriodictyon californicum*. Treatment with eriodictyol reduces TNFα, VEGF, intercellular adhesion molecule 1 (ICAM-1) and endothelial nitric oxide synthase in DR in rats and also prevents BRB breakdown [209]. Similar properties were found for hesperodine which also prevents AGE accumulation [180]. Green tea extract is widely studied for its beneficial properties protecting against brain ischemia, and it is a rich source of polyphenols. After 12 weeks of oral green tea treatment, diabetic rats restored low GFAP, ROS, glutamine synthetase, glutamate transporter and receptor levels in the retina [210]. At the same time, the expression of proinflammatory parameters (TNFα and VEGF) was significantly inhibited [211]. Chlorogenic acid is a polyphenol found in coffee, beans, potatoes and apples. It has beneficial effects on glucose metabolism [212,213]. It reduces VEGF expression and decreases BRB breakdown [214].

*Tinospora cordifolia* has a long history of use in Ayurvedic medicine. It contains many pharmacologically active ingredients, such as alkaloids, glycosides and steroids. Users maintain that it reduces DR due to its anti-hyperglycemic, anti-angiogenic, anti-inflammatory and antioxidant properties and prevents cataract and vascular changes [215,216]. This effect may be due to octacosanol, which is reported to downregulate VEGF gene expression by inhibiting matrix metalloproteinases and nuclear translocation of NFκB and reducing its DNA binding activity. Both glutathione and catalase levels increased in the treated group as compared to the diabetic group [215]. It decreases the overexpression of VEGF and alleviates the effects of high glucose [202]. Further substances examined for their protective effects in DR include curcumin [217,218], *Ginkgo biloba* leaf extract [219], zeaxanthin [220], lutein [221], fenugreek (*Trigonella foenum-graecum*) and resveratrol [118], all resembling each other in their mode of action.

With a different molecular target, berberine significantly decreased peroxisome proliferator-activated receptor-γ expression in diabetic retina [222]. *Ginseng* plants (*Araliaceae*), including North American ginseng (*Panax quinquefolius*) root extracts, have multiple pharmacological actions because of their diverse phytochemical constituents. Ginsenosides are its major bioactive factors. Ginsenosides or panaxosides are the derivatives of protopanaxatriol, a class of steroid glycosides, and triterpene saponins [223]. Bioactives of ginseng possess antioxidant properties, quenching free radicals, protecting low-density lipoproteins from oxidation and inhibiting lipid peroxidation [224].

Limitations of herbal formulas are worth mentioning, since most of them are not well established in human clinical research. A majority of them have been tried and justified in animal experimental models or in vitro pilot studies, which were translated to possible human applications. However, only a few of them became evidence based or clinically tried. Due to inevitable environmental factors, some herbal formulae may even contain some heavy metal contaminations. Unlike in the case of synthetic drugs, the effective molecules cannot always be clearly identified in the case of herbal medicines. Further, according to Bürgi’s law (see below Section 7) and principles of herbalism, plant-derived remedies rather have combined synergistic effects than just one useful molecule, so they cannot be simplified to a single mechanism.

#### 5.4.2. Endogenous Retinal Factors

There are several factors and processes associated with the neuron survival controlling oxidative status, autophagy and neuroinflammation in the retina [225]. These factors target different participants of the degeneration mechanisms and have already proved their therapeutic evidence in DR but are occasionally similar to those which play a role in glaucoma or age-related pathologies.

Erythropoietin (EPO) treatment reduced elevated IL-1 and TNFα expression in Müller cell culture and increased brain-derived neurotrophic factor (BDNF) expression in diabetic rat retinas [226,227]. Furthermore, EPO promotes ganglion cell survival in a rat model of glaucoma [228]. It ameliorates pro-survival signals involving Akt and reduces the loss of pericytes in the diabetic retina. EPO can reduce superoxide and other radicals, inducing glutathione peroxidase (GPX) and thus improving the balance between pro- and anti-oxidative factors and stimulating p-Akt. EPO treatment reduces pericyte dropout and also protects RPE cells against increased permeability. This effect is mediated by the downstream signaling of Janus kinase 2 and PI3K/Akt pathways [229].

Elevated pressure caused an increased pigment epithelium-derived factor (PEDF) and PEDF-R expression, which was related to Müller cells and ganglion cells in glaucoma. In addition, inhibition of PEDF expression resulted in increased apoptosis [230]. PEDF treatment has a reduced expression of several inflammation markers (such as IL-6, TNFα) in mouse models of DR [204]. Glucagon-like peptide 1 receptor agonist reduced microglia activation and TNFα expression in glaucoma models [231]. Furthermore, its topical administration was neuroprotective in a DR mouse model [232]. It also alleviates oxidative stress, apoptosis and autophagy in retinal cells of rats with induced diabetes [173].

Ciliary neurotrophic factor (CNTF) expression is altered in ganglion cells after glaucoma induction [233] and had a neuroprotective effect in glaucoma models of rat retinas [234,235]. Another similar compound, BDNF is expressed in RGCs and Müller glia [236] and causes delayed ganglion cell loss in glaucoma models [237] and attenuates abnormal microglial autophagy in DR [238]. Moreover, its role in AMD progression also has been investigated [239]. BDNF protects the neurons through (i) TrkB receptors, (ii) insulin responsive pathways and (iii) reduces systemic glucose levels locally in the retina. Inhibition of Müller cells and microglial connection attenuated microglia proliferation in a glaucoma model [240].

Autophagy flux decreased and apoptosis increased in ex vivo mouse retina explants under high glucose conditions, where application of the somatostatin analogue octreotide prevented cells from apoptosis. This protective effect of octreotide was demented by autophagy inhibition with chloroquine [241].

Besides different trophic factors, several endogenous antioxidant defence systems regulate the intracellular stress responses in the retina. When ROS production and antioxidants’ activity are imbalanced, oxidative injury and different retinal pathologies will occur [242]. ROS overproduction and decreased antioxidant defence engender damages of biochemical molecules and, if the repair mechanisms are inadequate, lead to apoptosis and neurodegeneration [242,243]. The main inducers of ROS production are hypoxia, metabolic defects, endoplasmatic reticulum stress and oncogenes and, on the other side, fundamental ROS scavengers are glutathione, NADPH, Nrf2, tumour suppressors and dietary antioxidants [244]. Antioxidants based on their origin could be exogenous (e.g., vitamin C, vitamin E, carotenoids) or endogenous. Depending on their activity, actions can be detected as enzymatic or nonenzymatic [245]. Furthermore, several studies have provided evidence for the lack of vitamin D triggering the pathogenesis of diabetes [246], including its accompanied syndrome DR [247]. This vitamin is a potent inhibitor of retinal neovascularization [247] through effectively decreasing VEGF levels [248].

Endogenous antioxidants are enzymes (e.g., superoxide dismutase (SOD), catalase, glutathione S-transferase, GPX, hem-oxygenase) or non-enzyme compounds (e.g., glutathione, melatonin, lipoic acid) and metal binding proteins (e.g., ferritin, transferrin) [249]. In the retina, the availability of different endogenous antioxidants decreases during metabolic retinal pathologies. In glaucoma, DR and aging models, increased ROS production [242,243,244,245,246,248,249,250,251,252,253] and decreased availability of different endogenous antioxidants has also been described in these pathologies [254,255,256,257,258,259].

Additionally, catalase, GPX, glutathione-reductase and copper-zinc SOD expression were downregulated in db/db mice [260]. Moreover, intravitreal SOD3 treatment prevented PEDF and parvalbumin expression and also reduced GFAP expression and ERG alteration [261]. Melatonin treatment reduced IL-6, TNFα, VEGF expression and apoptotic activity in STZ-induced diabetic retinopathy [262]. Photoreceptors showed lower catalase and higher lipid peroxidation in aging rat compared to younger rat retinas [263], and catalase-KO mice showed faster aging phenotype and impaired autophagy [264]. Decreased macroautophagy has been described in the aging retina. Furthermore, alterations in autophagy flux are also present in glaucoma and DR. Under high glucose conditions, autophagy is upregulated in Müller cells and rapamycin treatment could reset the autophagy and, as a consequence, VEGF expression remains at the baseline level [265].

#### 5.4.3. Synthetic Drugs

To manage DR by synthetic drugs, the major options are synthetic corticosteroids and macular targeting agents (See in Table S2 [135,136,137,138,144,147,148,149,266,267,268,269,270,271,272,273]). While macular target agents interfere with one or more pathways involved in the pathogenesis of DR, corticosteroids affect the anti-inflammatory pathway. Intravitreal injection of corticosteroids such as triamcinolone acetonide could provide more beneficial therapy than anti-VEGF. Besides the suppression of VEGF, it also inhibits prostaglandin production. Various cytokines are suspected to be involved in DR pathogenesis and have anti-apoptotic effects on retinal neurons [138,268,274,275].

Today, the most common molecular agents are the VEGF-inhibitors. Several clinical studies have demonstrated the advantage of intravitreal anti-VEGF injections (aflibercept, bevacizumab and ranibizumab) in reducing retinal neovascularization in patients with DR [137,147,148]. Bevacizumab binds and inhibits all isoforms of VEGF, but with a lower affinity, and has a longer half-life than ranibizumab [138,268]. Use of VEGF inhibitors in combination provides more benefits to therapy than standalone ones (see Section 7.2). Anti-VEGF inhibitors (e.g., bevacizumab, pegaptanib, ranibizumab, aflibercept) are also first-line medical options in DME treatment [276,277].

NMDA antagonists (such as Memantine) delay or halt ganglion cell death effectively, but the clinical use of NMDA antagonists is limited by their side effect profile, whereas NMDA receptors are widely presented and involved in different processes in the central nervous system. However, the combined usage could provide a promising strategy for DR or glaucoma therapy [81,147].

As we mentioned earlier, the main risk factors of DR are hyperglycemia, hypertension and dyslipidemia. They have a critical role in evolving a retinal inflammation state via different processes, e.g., oxidative stress, nitric oxide synthase dysregulation, AGE formation, renin-angiotensin system activation or inhibition of endogenous anti-inflammatory pathways. It is known that elevated levels of AGEs are present in pericytes, retinal blood vessels causing a blood–retinal barrier dysfunction and disruption of microvascular homeostasis. Disrupting the AGE, AGE receptor interaction is an important way to reduce the unwanted effect of accumulated AGEs. Furthermore, AGEs activate different intracellular pathways, NFκB and NADPH oxidase, ERK1/2, Janus Kinase signaling pathways, to increase ROS production and apoptosis of retinal neurons. Lower levels of the anti-apoptotic pERK and an elevated level of the pro-apoptotic marker pJNK protein are characteristic in aging and diabetic animals [85,278,279].

It has also been proved by numerous investigations that inhibition of the key regulator elements (TNFα [145,280], NFκB [135], PARP [281,282] or cyclooxygenase (COX) [283]) in diabetic-induced inflammatory response is effectively usable for delaying DR-associated vascular complications. COX-1 and COX-2 are key enzymes in the conversion of arachidonic acid to prostaglandin that are involved in inflammation. Through an elevated expression of VEGF, they cause vascular leakage and neovascularization [271], so COX inhibitors (acetyl salicylic acid, nepafenac) block inflammation indirectly. In the early stage of diabetic rat retinas, inhibition of COX-2 (but not COX-1) provides an effective management to reduce enhanced prostaglandin secretion. They also inhibited the diabetes-induced apoptosis of endothelial cells and pericytes, or the degeneration of retinal capillaries [136].

## 6. Glaucoma

Glaucoma is a heterogeneous group of progressive optic neuropathies characterized by typical optic nerve head alterations and the loss of RGCs. Among eye disorders, it is the second major cause of vision loss behind cataract and was the most common cause of irreversible blindness throughout the world in 2022 [30,86,87,284,285]. Glaucoma is expected to affect approximately 112 million people between 40 and 80 years of age by 2040 [18,30]. The incidence among the Caucasian population in the 40-year-old age group is approx. 2–3.5%, which increases with age, and in the 70-year-old age group, it is already 10%. Some types of glaucoma show familial accumulation, although the exact genetic background is still under intensive research. Only few animal models are at service for research: elevated IOP [286,287,288] and monosodium glutamate administration in young ages [289].

### 6.1. Risk factors and Disease Progression

Several risk factor can be considered when underlying causes are listed for glaucoma. Ocular perfusion pressure and optic disc haemorrhage, systemic hypertension, type 2 diabetes mellitus, smoking and/or high myopia and lipid dysregulation may all increase the risk for primary open-angle glaucoma (POAG) [30,108,285]. The most prominent modifiable risk factor however is the IOP exerted by the aqueous humour produced in the eye. The IOP varies individually, between 12 and 21 mmHg. Primary glaucoma is defined if there is no other ophthalmic or systemic disease besides glaucoma. In secondary glaucoma, the disease develops for a known reason [285]. In cases of normotensive glaucoma, a significant number of patients have shown optic nerve damage and vision loss, but not all ocular hypertensive patients develop glaucoma [118]. In glaucoma, the degeneration of RGCs and their axons in the optic nerve fibre layer accelerates, which is associated with damage to the optic nerve and causes visual field deficits. As soon as optic nerve loss and/or visual field loss develops as a result, glaucomatous excavation is detected and the diagnosis is established [30,285]. Retinal nerve fibres do not regenerate, so the potential target in the treatment of glaucoma aims to slow or prevent the progressive disruption of RGCs and a loss of their axons.

The progression usually stops if the IOP is lowered by 30–50% from its baseline [30]. Despite being on a combination of eye drops, patients may fail to achieve goal IOP reduction, and laser or surgical interventions are considered. Although high IOP is a risk factor for glaucoma progression, glaucomatous optic neuropathy can occur in individuals with a normal IOP [116]. Thus, interventions that focus solely on IOP reduction may not be beneficial for some glaucoma patients [129]. However, it is difficult or impossible to completely stop the progression of glaucoma, and many glaucoma patients experience progressive neurodegeneration [284]. It is therefore a potential target for new neuroprotective, IOP-independent therapeutic approaches [86].

### 6.2. Cellular Mechanisms

The death of RGCs occurs via apoptosis. Glutamate is the most abundant excitatory neurotransmitter in the retina. Excessive glutamate releases and the subsequent influx of Ca^2+^ via NMDA receptors has been implicated as the underlying cause of RGC death [118]. Several molecular mechanisms including neurotrophic factor deprivation, or the inhibition of inflammatory cascade, oxidative stress, mitochondrial dysfunction, glutamate-excitotoxicity, autophagy dysregulation, protein misfolding, ischemia and hypoxia. Each of these pathways may contribute to the etiology and progression of the disease group [18,86]. Neuroprotection in glaucoma is similar in concept to the treatment of central nervous system diseases including cranial trauma, stroke, amyotrophic lateral sclerosis, Parkinson’s disease, Alzheimer’s disease etc. [284,285]. The progression of the optic nerve and retinal neurodegeneration and pathogenesis of glaucoma have several hypotheses, but the neurodegenerative process often extends along the visual axis affecting the lateral geniculate nucleus and visual cortex [86]. Furthermore, these molecular events should not be considered as isolated but rather as a sequence of interconnected mechanisms where sustained oxidative stress acts as a key factor. Indeed, the imbalance between reactive species production and endogenous antioxidant defences sustains a vicious cycle that promotes chronic inflammation and creates a hostile environment for neuronal survival [86]. Retinal ischemia has been studied extensively because it has been proposed to be involved in a number of optic neuropathies such as anterior ischemic optic neuropathy, glaucoma, retinal and choroidal vessel occlusions, retinopathy of prematurity, DR and traumatic optic neuropathy [87]. The most fundamental neuropathy of all forms of glaucoma, characterised by a loss of the neuroretinal rim and a widening of the cup in the optic disc, is an injury due to the strangulation of optic nerve fibres in the optic disc, especially in the lamina cribrosa (LC). The increased pressure difference across the LC causes stress and strain on this structure, resulting in eventual compression, deformation and remodelling and therefore impedes anterograde and retrograde axonal transport within the optic-nerve fibres. Secondary stretching and thinning of LC can be found in progressive high myopia in association with an elongation and thinning of the parapapillary tissues that could lead to pronounced changes in the biomechanics of the optic nerve head and an increase in glaucoma susceptibility [30]. Although the mechanism of glaucoma is still largely unknown, oxidative stress, optic nerve ischemia and neuroinflammation were found playing certain molecular roles in the development of glaucomatous optic nerve degeneration [129]. Furthermore, new evidence suggests that glaucoma could be an autoimmune disease with progressive retinal degeneration caused by T-cells [290].

### 6.3. Molecular Considerations

There is evidence that dying neural cells create a toxic internal milieu which can affect healthy cells surrounding it. The essence of neuroprotection involves protection afforded to these healthy cells from damage. Therefore, there is an urgent need to improve therapeutic approaches to RGC damage that can be applied worldwide [87]. Interestingly, retina has its own growth and protective factor agent: the retinal glial cells and the RPE [145]. Neurotrophic factors are known to enhance the cell survival mechanisms and hence exert neuroprotective effects towards injury and degeneration. Müller cells and astrocytes are the two types of glial cells that express GFAP in the retina. Excessive GFAP levels are markers of metabolic stress in the retinal tissue. Moreover, a glial cell line-derived neurotrophic factor is also produced which has been shown to promote the survival of RGCs when introduced in different retinal injury rat models. [132]. RPE is also an important layer of retina which provides nutrition to other retinal layers and is thus indispensable for proper growth and survival. It produces several peptides (bradykinin, cortistatin, orexin, urocortin), and most of them seem to be neuroprotective [288,291]. RPE damage due to oxidative stress is one of the various factors responsible for pathogenesis leading to AMD [87].

The retina is particularly susceptible to oxidative stress due to its high consumption of oxygen, and this susceptibility increases with aging due to the physiological decrease of antioxidant defence mechanisms [292]. Clinical and experimental studies have reported that there is a significant correlation in human trabecular meshwork (TM) between oxidative stress, inflammation, increased IOP and visual field defects in glaucoma patients. As confirmed by previous studies, ischemia results in a reduced flow of blood to the retina and elevated levels of free radicals such as superoxide anion, hydroxyl radical and hydrogen peroxide, along with a progressive depletion in endogenous antioxidant enzymes including SOD, catalase and GPX, or antioxidants such as GSH. Ischemia results in deprivation of oxygen to tissues and metabolic substrates, ultimately affecting waste recycling. These processes lead to a homeostatic imbalance, exacerbating injury. Retinal ischemia has been studied extensively because it has been proposed to be involved in a number of optic neuropathies such as AION, glaucoma, retinal and choroidal vessel occlusions, ROP, DR and traumatic optic neuropathy [87]. Oxidative stress leads to TM cell dysfunction and increasing aqueous outflow resistance [86,131]. High levels of oxidative stress markers have been found in the aqueous humour of patients with POAG and Primary Angle Closure Glaucoma (PACG) and in the serum of pseudoexfoliation syndrome (PEX) patients [86]. In a clinical study, patients with POAG exhibited a low level of circulating GSH, suggesting a general decrease of antioxidant capacity [118]. Excessive free radicals cause oxidative stress, damaging lipids, proteins and DNA and ultimately resulting in cell death. Therefore, ischemia results in deprivation of oxygen to tissues and metabolic substrates, ultimately affecting waste recycling. These processes lead to a homeostatic imbalance, exacerbating injury.

Some important protective factors, however, are produced by retinal neurons themselves. Brn3b is a major transcription factor that belongs to the mammalian POU family. It is a key regulatory marker of RGCs and is known to play an important role in RGC development and survival. BCL-2, an anti-apoptotic gene critical for the inhibition of programmed cell death [293,294,295], is known as an essential controller of PRs (rods, cones) and RGCs death in degenerated retinas [296,297,298].

### 6.4. Therapeutic Approaches

Currently, glaucoma cannot be cured, but with early detection and proper treatment of the disease, the rate of progression can be slowed and vision can be preserved [284,285]. Lowering IOP is the primary evidence-based therapeutic strategy including laser trabeculoplasty, glaucoma filtration surgery and antiglaucoma medications [30,284]. POAG is the most common type, and it differs from normal tension glaucoma in that in the former, elevated IOP occurs. Left uncontrolled, glaucoma has irreversible devastating visual consequences, therefore, it is called “the sneaky thief of sight” [134].

#### 6.4.1. Some Herbal Remedies

Early pharmacological treatments of glaucoma relied on herbal remedies and diet, but the preparation of these medicines were empirical [129]. One of the first herbal-based active compounds used for glaucoma was pilocarpine—a parasympathomimetic alkaloid extracted from *Pilocarpus jaborandi*. It is still available for glaucoma management. It causes ciliary muscle contraction which facilitates the aqueous humour outflow through the TM [129]. Forskolin eye drops (1%) in India were applied in POAG to reduce IOP and visual field defects with efficient improvement of optic neuropathy. Hypothetically, Forskolin eye drops might be a safe alternative to β-blockers in glaucoma patients having concomitant asthma [89].

However, use of herbal medicines is not without potential side effects. Edington et al. explained in their case report that *Hypericum perforatum* L. may affect IOP control, because it is known to interact with several drugs, including β-blockers, as it induces the cytochrome P450 system and causes faster drug metabolism. Studies have previously confirmed high cytochrome P450-dependent monooxygenase activity in the ciliary body, iris and RPE in bovine and human eyes [30,299]. They reported the case of a POAG patient with suboptimal IOP control, which improved on discontinuation of the *Hypericum perforatum* product. It is, therefore, possible that the herbal compounds could have the same receptorial and enzymatic effect in the eye as it does elsewhere in the human body [299].

#### 6.4.2. Synthetic Glaucoma Drugs

Besides herbal medicines, other pharmacological agents are in the toolset of preventing or circumventing glaucoma. Current treatment typically involves topical ocular hypotensives as first line therapy, and the six major drug classes are as follows: β-adrenergic blockers, α2-adrenergic agonists, prostaglandin analogues, carbonic anhydrase inhibitors, rho kinase inhibitors and cholinergic agonists (See in Table S2 [136,139,140,141,150,266,267,270,273,300,301,302,303,304,305,306,307,308,309,310,311,312,313,314,315,316,317,318,319,320,321,322,323,324,325,326,327,328,329]). These work to lower IOP by preventing aqueous humour build up in the anterior chamber via decreasing aqueous humour production from the ciliary body or increasing aqueous humour outflow through the trabecular meshwork or uveoscleral pathway [5,45]. For example, brimonidine is an α2-adrenergic agonist and is used to lower IOP and ocular hypertension in open-angle glaucoma. Several studies demonstrated that brimonidine could promote the survival of RGC [141,308,330]. Acheampong et al. proved that topically applied brimonidine widely distributes into the posterior segment of monkey and rabbit eyes after single and multiple dosing and that intraperitoneal administration of brimonidine also results in the significant availability of brimonidine in the posterior segment of rat eyes [330]. α2 receptors activate multiple pathways for instance the activation of intracellular kinases enhance cell survival, the induction of anti-apoptotic genes such as BCL-2, neuronal survival factors such as basic fibroblast growth factor and the inhibition of glutamate release and calcium influx into cells [141,308]. Another α2 adrenoreceptor agonist is apraclonidine which is used topically and can also decrease IOP in glaucoma patients by increasing trabecular outflow without cardiovascular side effects [267].

The β-blockers effectively lower IOP to decrease aqueous inflow and increase uveoscleral outflow during the day but not during the night [331]. Non-selective beta-adrenergic antagonists (carteolol, levobunolol, metipranolol, timolol) are mainly used in combination. It is important to know that they should not be given to patients with cardiac or pulmonary insufficiency [267,316,317]. Betaxolol is a β1-selective adrenergic antagonist, but it has less effects on cardiac and pulmonary functions than non-selective β-blockers. However, special attention has to be given when betaxolol is prescribed to patients with asthma.

Prostaglandin analogues are currently used as the most powerful agents to reduce IOP [307]. Several studies have demonstrated that the regulation of matrix metalloproteinase and remodelling of the extracellular matrix change the permeability and cause enhanced uveoscleral outflow with minor effects on trabecular outflow and aqueous flow [307]. However, remarkable effects on the cardiovascular system are characteristics for them [307,332]. An intracameral implant, brimatoprost is used to reduce IOP for up to four months after insertion in patients with glaucoma [333]. Preclinical and animal studies have demonstrated that tafluprost also promoted efficacy and safety in the treatment of glaucoma [322].

Another therapeutic approach is the application of carbonic anhydrase inhibitors to inhibit the activity of carbonic anhydrase 2 and HCO_3_^−^ production in the non-pigmented ciliary epithelium, thus reducing aqueous humour formation. Acetazolamide and methazolamide are approved as oral medications, but topical eye drops are better tolerated, such as dorzolamide or brinzolamide [267,300,311,312,313,319]. In glaucoma, SOD, catalase, GPX and melatonin levels significantly decreased in rat retinas after 6 weeks of hyaluronic acid treatment [258]. GPX and catalase levels also decreased after sodium hyaluronate induction of ocular hypertension in rat retinas [259]. Furthermore, downregulation of glutathione was also described in glaucomatous eyes [261]. SOD and GPX levels decreased, and IL-6 and ICAM-1 levels increased in diabetic rat retinas [334]. Rapamycin treatment was neuroprotective by downregulating caspase-3 expression and inhibited TNFα, ROS and NO expression in different glaucoma models [335].

Rho(ρ)-kinase (Rho-associated coiled-coil containing protein kinase; ROCK), a member of the serine-threonine protein kinases, involve various physiological functions such as chemotaxis, neural growth and gene expression [336]. Most ROCK inhibitors reversibly compete with ATP, because their main target is the ATP-binding site of the kinase in its active conformation [337]. ROCK inhibitors (ripasudil, netarsudil, fasudil) have demonstrated efficacy in reducing IOP in animal models and humans [273,320,337,338,339,340,341,342]. Streptozotocin-induced diabetic models confirmed activation of the Rho/ROCK pathway in retinal microvessels, significantly reducing ICAM-1 expression and the number of damaged endothelial cells. The administration of oral ROCK inhibitor delayed RGC death [341,342]. The classic ROCK inhibitors, fasudil, is isoquinoline-derived and also inhibits other kinases including protein kinase C, protein kinase A and myosin light chain kinase [343]. Completed clinical trials verified the safety and effective application of 0.4% ripasudil to lower IOP [344,345,346]. However, usage with beta-blockers and prostaglandin analogues could provide more beneficial pressure-lowering effects [347].

## 7. Combination Treatments for the “Big Three”

Emil Bürgi, over 100 years ago, claimed that the combination of at least two different therapeutic substances or medications with equal or similar activities can lead to addition; on the other hand, when their points of action are different, they can produce greater synergy or coalism than the simple additive sum of their individual mechanisms (Bürgi’s law) [87,284]. It is important to remember that plant extracts used at that time for medications contained not a pure compound but other compounds that are present in order to have a therapeutic effect [88]. Experimentally proven pharmacological effects can be demonstrated by *Curcuma longa* L., which has limited oral absorption and systemic availability [90,91]. Piperine, a component of *Piper nigrum* L., is among the bioavailability enhancers used for this purpose. This agent, when used together with curcumin from *C. longa*, improves its bioavailability by 2000% [91].

In order to be effective in cures, the events which have to be avoided or prevented are (**1**) (para)inflammation and/or glial cell activation, (**2**) ischemia and related ROS and VEGF accumulation, (**3**) apoptosis and/or autophagy of nerve cells and (**4**) elevation of ocular perfusion pressure and/or IOP. Since the underlying causes in the case of the “big three” are similar, only the potential of the individual harming factors is different, and often the treatment options are also similar. Making things worse, in the case of aging, diagnosis is often difficult to tell apart from the other conditions, since patients may report vision changes in one of their eyes for a shorter or longer period of time, and characteristically, they describe central blurring in their vision and bending or waviness in straight lines and may report increasing difficulty reading print [348], which can be the hallmarks of the other two disorders at the beginning. Only detailed diagnostic examinations can confidently differentiate the disorders. In light of the above, we classified the combination treatments into the following categories: (i) device-assisted minimally invasive therapy plus drug application; (ii) antiangiogenic treatment plus drug application; and (iii) internal neuroprotective factors and drugs combined.

### 7.1. Device-Assisted Minimally Invasive Therapy plus Drug Application Approach

Since seriously invasive device-based therapies such as thermal laser photocoagulation have their limitations, therapies with other mechanisms of action are also solicited for. Possibilities for combination therapy are photodynamic therapy combined with anti-inflammatory drugs consisting of either corticosteroids or non-steroidal anti-inflammatory drugs. One of these treatments, photodynamic therapy combined with verteporfin administration, was recently evaluated by the Treatment of Age-Related Macular Degeneration with Photodynamic Therapy study group. The results showed that at 1 year, there was a 15% increase in eyes that retained better visual acuity than those that received a placebo. At 2 years, this ratio was 32% [349]. From the underlying causes listed above, this approach targets **3** and **1**.

Adjunct to intravitreal pharmacotherapy (corticosteroids, anti-VEGF therapy etc.), other device-assistant evidence-based treatments are also available during the management of DR. These include laser photocoagulation and vitreoretinal surgeries [350,351,352]. Laser photocoagulation techniques (peripheral retinal laser photocoagulation, focal macular laser photocoagulation, grid photocoagulation) offer a reliable treatment option in proliferative retinopathy, macular oedema and retinal detachment. This type of laser surgery targets the newly formed and abnormal leaking vessels [353,354]. In clinical practice, another surgical technique is vitrectomy which serves for the treatment of vitreous haemorrhages, combined/tractional retinal detachment, macular heterotopia and fibrovascular proliferations [352,355].

In the case of glaucoma, the only proven and generally accepted treatment to reduce the risk of further progression of glaucomatous optic neuropathy is to lower IOP. Reduction of IOP is achieved by drug treatment, laser therapy or surgery. In eyes with an open anterior chamber angle, drug treatment could be augmented by, or in some cases replaced by, laser therapy (laser trabeculoplasty) to the trabecular meshwork, in particular if the target IOP is not achieved by use of drugs (particularly in poorly compliant patients). Independent of concurrent drug treatment, laser intervention can reduce the IOP by a few additional mm Hg. The good safety profile of laser trabeculoplasty is combined with its fairly low efficacy. If the IOP-lowering effect is not sufficient, incisional glaucoma surgery has to be done, usually under local anaesthetic but occasionally under topical anaesthesia. In patients with poor compliance or those intolerant to drug treatment, incisional surgery can also be conducted as the first step in the treatment of glaucoma. A panoply of surgical antiglaucomatous procedures have been developed in the past decade. Creating an additional outflow pathway from the eye for the aqueous humour, all surgical techniques (e.g., trabeculectomy) risk reduced long-term success secondary to fibrosis around the subconjunctival exit point of the fistula. During and after surgery, antimetabolites are applied to the surgical site to decrease the fibrotic response and to keep the fistula site open. Glaucoma implant drainage devices are another surgical option and act by channelling the aqueous humour through a tube out of the eye into the subconjunctival space. These devices are similarly effective in lowering IOP to trabeculectomy [356]. Minimally invasive glaucoma surgery, compared with standard trabeculectomy, has fewer side-effects but lower efficacy [357]. Similarly, trabeculectomy versus non-penetrating surgeries (e.g., deep sclerectomy, viscocanalostomy, and canaloplasty) is more effective at reducing the IOP but has a higher risk of complications. Treatment of congenital glaucomas is mainly surgical [30].

Recently, there has been increasing interest in the promising usage of stem cell therapy to achieve ocular tissue (RGC, RPE and photoreceptors) repair and/or regeneration. For the big three, only a limited number of studies based on stem cell-based therapies are currently completed worldwide (according to clinical trials.gov). Most of these trials are in the recruiting or unknown status. There are different methods of stem cell therapy. First, pluripotent stem cells can be used for the forward-moving development process to induce cell proliferation into the tissue of interest. Second, partially differentiated cells can be made backward in their developmental lineages and then redirect them onto the desired end tissue [358,359]. Their efficacy is dependent on the success of adult cells to return to the pluripotent stage, using molecular manipulation by retroviruses and lentiviruses to carry genes encoding transcription factors. This novel approach of glaucoma therapy is also represented in the recent scientific literature with the advances of the modified induced pluripotent stem cell technology. In cases of glaucoma that require tissue repair and regeneration of trabecular meshwork cells, RGCs are promising. Some modifications are required so that stem cells do not only have to differentiate into RGCs but also have to grow axons that are able to reach the retinal target areas in the central nervous system. The factors which guide the axons of the developing RGCs in order to reach the optic chiasm and stimulate synapse formation in the superior colliculus and the lateral geniculate nucleus still need to be better explored [360].

### 7.2. Anti-Angiogenic Injection Therapy and Drugs

Anti-VEGFs are classified as antibodies for VEGF and VEGF receptor-1 and -2 antibodies or alternatively, VEGF receptor tyrosine kinase inhibitors. Among combination therapies, anti-VEGF combination with sustained-release corticosteroids appears promising [87]. Additional candidates are natural products, but these may also serve as preventive measures. For each medication, there are several and severe adverse effects, but natural products have a potency as AMD, DR or glaucoma drugs, since they have been used as culinary materials and/or traditional medicines for a long time (see above in the relevant sections). In general, AMD drug candidates from natural products are more effective at treating early and intermediate AMD [361]. From the underlying causes, this approach fights **2** and **3**.

It has been found that non-steroidal cyclooxygenase inhibitor nepafenac [193,362,363], salicylates [364] and reactive nitrogense species decomposition catalysts [365] inhibited RGC degeneration in DR. This offers an excellent opportunity to supplement bevacizumab treatment with drugs. In the case of patients with moderate visual loss or worse, aflibercept may be considered. Both the clinical trials and the laboratory investigations agree that besides the efficacy of the drug, the manner of administration may be the clue to treat retinal complications, since many of the drugs do not cross the BRB, but intraocular injection may be effective. This approach targets mechanisms **2** and **4**.

In addition, the need for safe sustained delivery devices has emerged recently. The ultimate success of the delivery system will depend on efficacy relative to eye drop dosing, safety and patient acceptance. Cautious development efforts are warranted, considering prior failed approaches for sustained glaucoma drug delivery [366].

### 7.3. Internal Neuroprotective Factors and Drugs

Peptides acting through the insulin-like growth factor pathway attenuated the diabetes-induced apoptosis of retinal neurons and mitochondrial metamorphosis in RGCs. Consistent with these effects, it decreased cleaved caspase-3 and p-STAT3 levels. Rosiglitazone is an anti-diabetic drug in the thiazolidinedione class of drugs and works as an insulin sensitizer by binding to peroxisome proliferator-activated receptors in fat cells and making the cells more responsive to insulin. After treatment with rosiglitazone in experimental DR, the retina thickness and the RGC numbers were significantly greater than in control animals. Consequently, rosiglitazone might be used to prevent retinal neuronal damage in diabetes [367].

Targeting another pathway, with the combination of PACAP and the poly(ADP-ribose) polymerase inhibitors, good protection could be achieved in rat experimental DR models. This combination was useful in the case of diabetic animals with elevated systemic blood pressure (spontaneously hypertensive rats). Interestingly, this protection, provided by the distinct pathways **1**, **3** and **4** listed above, seemed fully additive [368]. However, not all the combination treatments are successful. PACAP treatment alone and an enriched environment alone are neuroprotective, but their combination is not additive [369]. This situation may be due to overlapping intracellular protective pathways of the two protective mechanisms.

## 8. Conclusions

**Figure 2:**
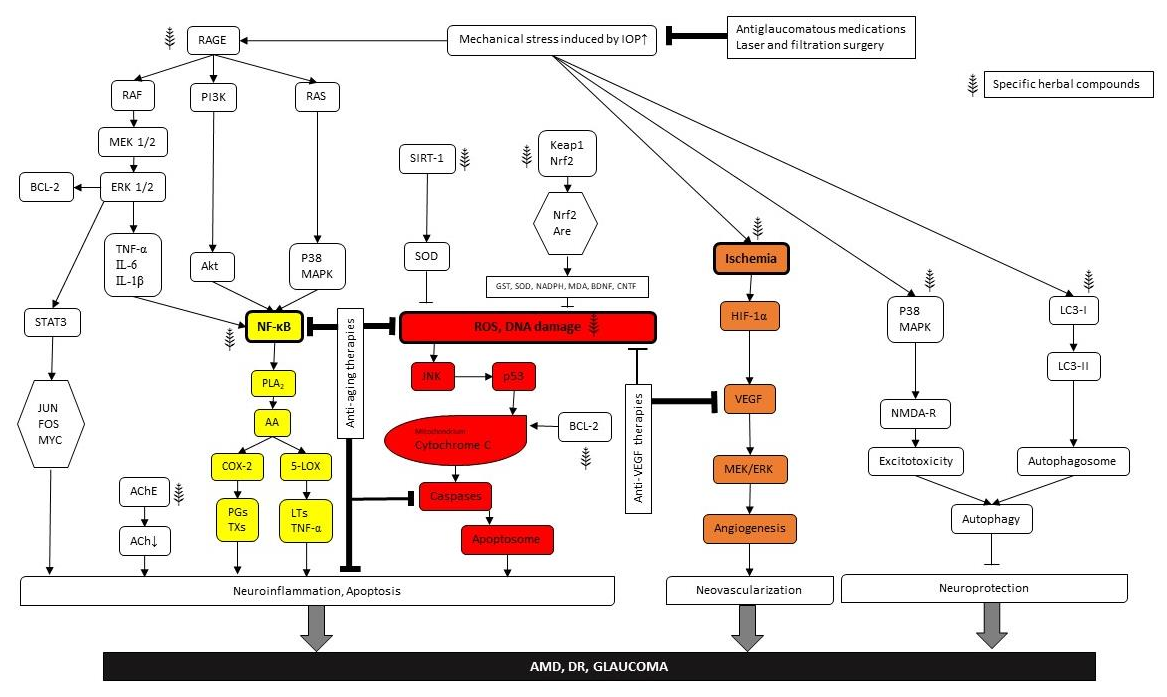
Pathways presenting the evolution and therapeutic opportunities of the “big three” (AMD, DR, glaucoma). Arrows indicate the intracellular signal transduction pathways. Bold frames demonstrate the main nodes. Color coding means as follows: yellow—(para)inflammatory processes, red—DNA damage and orange—anti-VEGF therapies. Herbal signs demonstrate phitoremedial intervention sites.

As for conclusions, we can state that there are common causes for diseases and common targets for protection in the case of the “big three”, age-related retinal degeneration, diabetic retinopathy and glaucoma. The intertwined molecular pathways are presented in Figure 2 as well as possible intervention possibilities. We identified three major nodes within the metabolic mesh at which effective interventions can be initiated: NFkB induction due to (para)inflammatory processes, ROS (DNA damage) reduction and anti-VEGF therapies. The first two lead to reduction of apoptotic damage, while the third one effectively inhabits neovascularisation, preventing retinal detachment and consequent vision loss. Besides direct targeting of the metabolic nodes of this network, we provided some proof that a healthy lifestyle can be an important factor in preventing metabolic retinal disorders. Furthermore, herbal medicines can be applied both for prevention and treatment purposes. We may also utilize the internal substances of our own body, particularly neuropeptides and trophic factors as well as synthetic drugs. There are a number of possibilities to combine these treatment options. However, it is important to note that we should target at least two of the four major harm-causing processes, namely (para)inflammation and/or glial cell activation, ischemia and related ROS and VEGF accumulation, apoptosis and/or autophagy of nerve cells and elevation of ocular perfusion pressure and/or IOP. This way, interventions maybe rather efficient. It might also be useful to seek repositioning of existing drugs and use them for the cure of the other related conditions. Targeting two distinct intracellular pathways may result in additive protection (Figure 2).

Considering that vision loss threatens approximately 20% of the ever-growing and aging population of the world, safe, affordable and easily available therapies for the “big three” are badly needed. Some of the potential avenues to achieve these goals were outlined in this review.

## Data Availability

No new data were created or analysed in this study. Data sharing is not applicable to this article.

## Supplementary Materials

The following supporting information can be downloaded at: https://www.mdpi.com/article/10.3390/ijms24108728/s1.

## Abbreviations

AGEs	advanced glycation end products
AMD	age-related macular degeneration
AION	anterior ischemic optic neuropathy
AREDS	Age-Related Eye Disease Studies
BRB	blood–retinal barrier
BDNF	brain-derived neurotrofic factor
CNTF	ciliary neurotrophic factor
COX	cyclooxygenase
DR	diabetic retinopathy
DME	diabetic macular edema
ERG	electroretinogram
EPO	erythropoietin
GFAP	glial fibrillary acidic protein
GPX	gluthatione peroxidase
IL	interleukin
ICAM-1	intercellular adhesion molecule 1
IOP	intraocular pressure
NVU	neurovascularunit
NMDA	N-methyl-D-aspartate
NFκB	nuclear factor κB
PEDF	pigment epithelium-derived factor
PACAP	pituitary adenylate cyclase-activating polypeptide
PR	photoreceptor
POAG	primary open-angle glaucoma
ROS	reactive oxygen species
RGC	retinal ganglion cells
RPE	retinal pigment epithelium
ROP	retinopathy of prematurity
ROCK	Rho-associated coiled-coil containing protein kinase
PEX	serum of pseudoexfoliation syndrome
SST	somatostatin
SOD	superoxid dismutase
TM	trabecular meshwork
TNF	tumor necrosis factor
VEGF	vascular endothelial growth factor
