# Supplementary material for: Three Major Causes of Metabolic Retinal Degenerations and Three Ways to Avoid Them
Source: Int J Mol Sci. 2023 May 13;24(10):8728. doi: 10.3390/ijms24108728 (PMC10218427; doi:10.3390/ijms24108728)
Supplement: Supplementary file 1 [file ijms-24-08728-s001.zip › TableS1.pdf]

| Scientific name, botanical family | Phytochemicals and marker molecules | Structure of main marker molecules | Pharmacobiologic properties | Adverse effects, limitations | Target retinal diseases | References |
|---|---|---|---|---|---|---|
| ***Allium cepa* L. (Amaryllidaceae)** | Glycosides and flavonoids (**quercetin**, quercetin 3,4′-diglucoside, quercetin-4′-glucoside, myricetin, kaempferol), organosulphur compounds (thiosulphimate, cepaene, S-methyl cysteine sulfoxide, allicin, diallyl-trisulfide, S-allyl cysteine) | 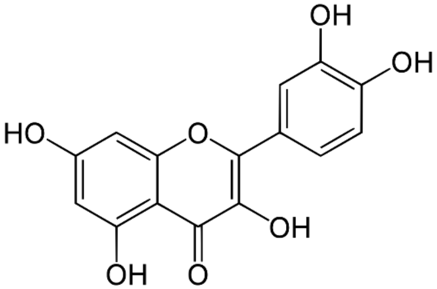 | Neuroprotection against brain ischemia-reperfusion via Wnt/catenin pathway<br>Inhibits the Caspase-3 activity, Cyt-C and protects RPE cells<br>Antioxidant effect<br>Inhibits VEGF<br>Increase in the GDNF expression which will further promotes cell survival<br>Significant increase in the expression of antiapoptotic genes Brn3b and Bcl-2<br>Glibenclamide-analogueous antidiabetic activitiy, increasing GLUT-4 receptor expression, PI-3-K/Akt signaling pathway to regulate glucose transport<br>Downregulating anti-inflammatory mRNA expression<br>Increasing the levels of TNF-α and IL-6 and IgM and IgG, preventing the immunosuppressive macrophage activities | Hemolytic anaemia in canines<br>No toxicity data in humans, dietary risks (e.g. pesticides) are negligible | AMD<br>DR<br>Glaucoma | Alok et al. (2014)<br>Anand et al. (2014)<br>Ikonne et al. (2020)<br>Kumar et al. (2020)<br>Zhao et al. (2021)<br>Chakraborty (2022)<br>Pokkalath et al. (2022) |
| ***Ananas comosus* (L.) Merr. (Bromeliaceae)** | **Bromelain** | 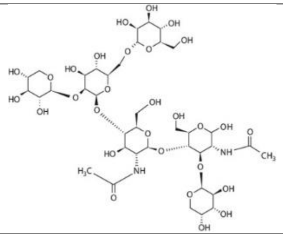 | Helps to reduce vitreous opacities and hemorrhage<br>Anti-inflammatory agent: reduces IL-1β, IL-6 and TNF-α secretion<br>Suppression of bFGF, FGF-2, VEGF, angiopoetin-1 and 2, COX-2, MMP-9, AP-1, NF-κB activation<br>Cysteine protease enzyme activity (proteolysis and hydrolysis) | Recognized as safe<br>The plant stem contains the most enzyme, but has higher risk of individual allergy and hyperglycemia | DR | Rathnavelu et al. (2016)<br>Takeuchi et al. (2020)<br>Chiosi et al. (2022) |

| | | | | | | |
|---|---|---|---|---|---|---|
| ***Anisodus tanguticus* (Maxim.) Pascher (Solanaceae)**<br>TCM: 山莨菪 (shān làng dàng)<br>TTM: ཐང་ཕྲོམ་ནག་པོ་. ཐང་ཕྲོམ་ནག་པོ་ (thang phrom nag po) | Tropane alkaloids (anisodamine, **anisodine**, hyoscyamine, scopolamine, tropine, apoatropine, trichlorophenyl butyryloxytropane) and non-tropane alkaloid (cuscohygrine) | 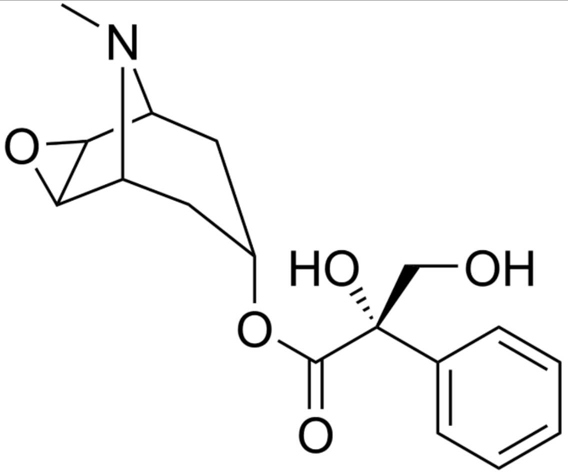 | Anticholinergic effect, antidote for organophosphate poisoning<br>Microcirculation improving properties, increasing retinal blood flow and oxygen supply to the retinal tissues<br>Prevent retinal lipid peroxidation<br>Downregulating the expression of PAI-1 and TF via blocking NF-κB-signaling pathway<br>Protective in DR: Activate α7nAChR, that decreases VEGF expression and NV<br>Antiapoptotic: Inhibit the production of TNF-α and other cytokines (IL-β and IL-8)<br>Downregulate VPO-1 and inhibit JNK1/2 phosphorylation therefore prevent oxidative stress-induced damage and cell apoptosis | The plant is toxic due to its tropane alkaloids and can cause poisoning when it is ingested or used inappropriately | DR | Behl & Kotwani (2017)<br>Guo et al (2022) |
| ***Arctium lappa* L. (Asteraceae)**<br>TCM: 牛蒡 (Niúbàng) | **Arctiin**, tannin, arctigenin, β-eudesmol, caffeic acid, chlorogenic acid, inulin, trachelogenin 4, sitosterol-β-D-glucopyranoside, lappaol, diarctigenin | 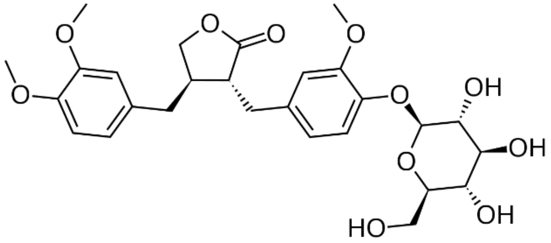 | Reduces retinal edema and retinal detachment<br>A2E accumulation is significantly suppressed in a concentration-dependent manner<br>Inhibition of iNOS expression, NO production and NF-κB pathway<br>Suppression of pro-inflammatory cytokine expression<br>Activation of antioxidant enzymes and scavenging of free radicals | Generally safe, but contact dermatitis can occur by external use its oil<br>One literature reports case of anaphylaxis | dAMD<br>DR | Chan et al. (2011)<br>Kim et al. (2020)<br>Pokkalath et al. (2022) |
| ***Artemisia annua* L. (Asteraceae)**<br>TCM: 青蒿 (qīnghāo) | **Artemisinin**s: Artemisinin (qinghaosu, 青蒿素), Dihydroartemisinin (DHA), Arthemeter, Artheeter, Artesunate, Artemisone | 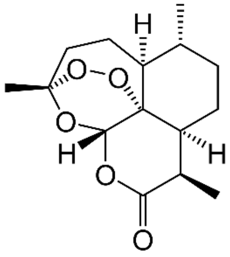 | Inhibition of retinal and corneal angiogenesis: Inhibition of HIF-1α, VEGF, Ang-1, VEGFR, PDGFR, upregulating angiogenesis inhibitors: flt-1 and KDR/flk-1-receptors<br>Decreasing TLR4, TLR9, TNF-α expressions, also suppressing NF-κB activation<br>Prevention of RPE cells from oxidative stress via the MAPK/CREB pathway | Generally well tolerated<br>Minor side effects can be nausea, vomiting, anorexia, and dizziness<br>Potentially severe adverse events include prolongation of the QTc interval and cardiac arrhythmias | AMD<br>DR | Lu & Xie (2019)<br>Chiosi et al. (2022) |

| | | | | | | |
|---|---|---|---|---|---|---|
| ***Astragalus membranaceus*** **Fisch. ex Bunge (Fabaceae)**<br>TCM: 黄芪 (huáng qí) | Polysaccharides (astragalans I, II and III), saponins (**astragalosides I**–VIII and isoastragalosides I and II), flavonoids, isoflavonoids, sterols, amino acids, volatile oils, and trace elements | 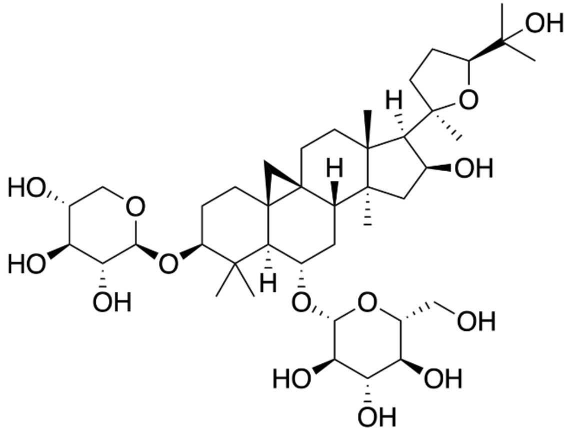 | Reduce retinal ganglion cell apoptosis<br>Decrease phosphorylation of ERK1/2<br>Inhibit activation of NF-κB and various cytokines<br>Downregulate the expression of enzyme aldose reductase (polyol pathway)<br>Prevent inflammatory responses (LPS-induced cytokine secretion) in retinal microglia and inhibits microglia activation<br>Downregulate p38 MAPK signaling<br>Inhibition of AGE-induced overproduction of pro-inflammatory cytokines (IL-1β, TNF-α)<br>Reducing the hyperglycemia-induced overexpression of VEGF in retinal Müller-cells<br>Significantly lower IOP | May be safe, some uncommon possible side effects with oral use include rash, itching, nasal symptoms, or stomach discomfort<br>Can be toxic to fetus according to some animal studies, thus not recommended in pregnancy | DR | Anand et al. (2014)<br>Behl & Kotwani (2017) |
| ***Berberis kansuensis*** **Schneid. (Berberidaceae)**<br>TCM: 小檗皮 (Xiǎo bò pí)<br>TTM: སྐྱེར་པ (Skyer pa) | **Berberine**, Berberamine, magnolia alkaloid, palmatine, jatrorrhizine | 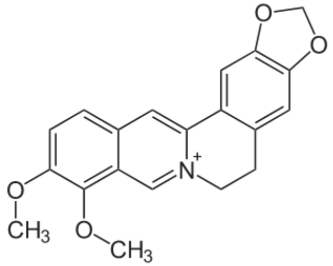 | Protective effect on retina: inhibition of PKC-β, VEGF and HIF-1α<br>Block the voltage-dependent $Ca^{2+}$-channel and receptor-dependent $Ca^{2+}$-channel<br>Regulating the retinal vascular endothelial homeostasis<br>Regulate the expressions of P-TEFb (CDK9 and CyclinT1) in the retina<br>Significantly reduce blood glucose and blood lipids | Severe side effects and toxicity are not known, but not fully elucidated, yet | DR | Gao et al. (2021) |
| ***Bixa orellana*** **L. (Bixaceae)** | 6,6′-di-apo-carotenoid (**9′-cis-Norbixin,** E160b) | 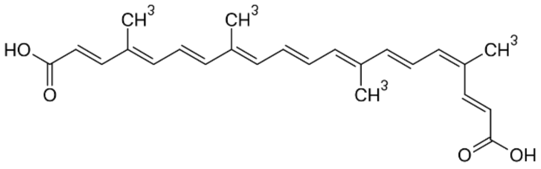 | Protects retinal tissues and photoreceptor cells<br>Prevents the reduction of rod and cone photoreceptor electrical activity measured by scotopic and photopic ERG amplitude)<br>Reduced the uptake of A2E by RPE cells and slowdowns the progress of A2E accumulation<br>Reduces the production of VEGF and inflammatory cytokines (IL-1, TNF-α, IL-6)<br>Reduces ROS production to oxidative stress | Acceptable daily intake 0.3 mg/kg/day | iAMD | Camelo et al. (2020) |

| | | | | | | |
|---|---|---|---|---|---|---|
| ***Boswellia sacra*** **Flueck. (Burseraceae)**<br>TCM: 乳香 (rǔ xiāng) | Boswellic acids [**Acetyl-11-keto-β-boswellic acid** (AKBA), β-boswellic acid (β-BA), α-boswellic acid (α-BA) etc.], lupeolic acid, betulinic acid, incensole acetate, incensole oxide, isoincensole oxide, isoincensole acetate | 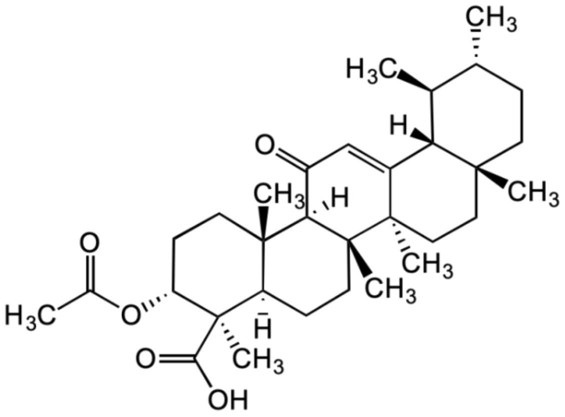 | Reduce retinal NV<br>Inhibit VEGF expression and VEGFR-2 phosphorylation in the retina<br>Activate SHP-1 and inhibits STAT3 phosphorylation in the retina<br>Do not affect retinal cell survival or retinal function significantly<br>Inhibits proliferation, migration and tube formation of RPE cells<br>Increased level of Nrf2 and HO-1 after long-term treatment<br>Effectively reduce MBP levels in CSF and restore MBP levels in brain<br>Decreased Caspase-3, Bax, and increased Bcl-2 levels<br>Significantly decrease the pro-inflammatory cytokine TNF-α in brain and blood serum<br>Amelioration of oxidative stress markers (AChE, MDA, nitrites)<br>Suppress NF-κB activation, human leukocyte elastase, and consequently chronic inflammation<br>Target molecules: 5-LOX/COX, mPGES-1, CatG), amyloid plaques, and neurofibrillary tangles<br>Promising anti-inflammatory drug candidate for treating neurodegenerative diseases, cerebral oedema<br>Inhibit glutamate excitotoxicity and oxidative neuronal injury | Well tolerated<br>Without side effects | AMD<br>DR<br>ROP | Lulli et al. (2015)<br>Rajabian et al. (2019)<br>Siddiqui et al. (2021)<br>Upadhayay et al. (2022) |
| **Brazilian Green Propolis** | **Caffeoylquinic acid** derivates, flavonoids (quercetin, kaempferol), polyphenols (gallic acid, Caffeic acid, caffeic acid phenethyl ester (CAPE), apigenin and artepellin C) | 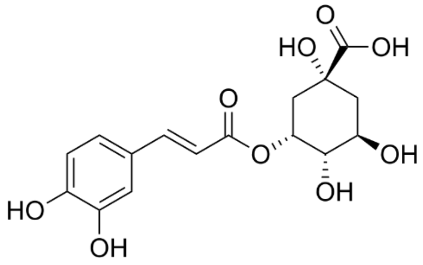 | Neuroprotective effect by reducing retinal damage<br>Reduced oxidative stress<br>Preventing VEGF-induced angiogenesis<br>Reduction of inflammatory processes by PAK1 inhibition and by inhibiting the inflammasome<br>Promotes immunomodulation of the NF-κB/TLR4 system, with reduction of interleukins | Considered as safe<br>No known significant drug interaction | AMD<br>DR<br>ROP | Anand et al. (2014)<br>Silveira et al. (2021) |

| | | | | | | |
|---|---|---|---|---|---|---|
| ***Camellia sinensis* (L.) Kuntze, (Theaceae)** | Alkaloids (caffeine, theobromine), Flavanols, Flavonoids, Polyphenols, Tannins [catechins e.g. **epigallocatechin-3-gallate** (EGCG), epigallocatechin (EGC), epicatechin-3-gallate (ECG), epicatechin (EC)], Minerals | 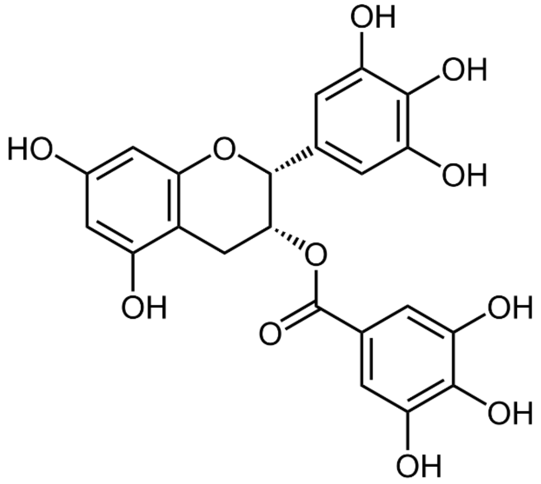  | Suppress VEGF-mediated corneal NV<br>Inhibits NV and MMP-2 activity<br>Prevents the formation of acellular capillaries and vascular leakage<br>Significant increase in the a- and b-wave amplitudes of ERG<br>Decrease the apoptotic pathways, reduce Müller cell apoptosis, ROS production and restores glutathione expression<br>Neuroprotective effect on ON trauma<br>Decrease NO expression and upregulation of Akt/PI3K pro-survival pathway<br>Ameliorate ischemia-induced RGC degeneration and prevent RGC function impairment through antioxidant and anti-inflammatory pathways | Considered as safe and usually well tolerated<br>Because of the caffeine content, is contraindicated in pregnancy, and can cause minor side effects<br>Very rarely can cause liver toxicity | AMD<br>DR<br>Glaucoma and OHT | Alok et al. (2014)<br>Karhanová et al. (2015)<br>Adornetto et al. (2020)<br>Ikonne et al. (2020)<br>Tseng et al. (2021)<br>Pokkalath et al. (2022) |
| ***Cannabis sativa* L. (Cannabaceae)**<br>TCM: 大麻 (Dàmá) | Δ-9-tetrahydrocannabinol (THC), **cannabidiol** (CBD), cannabigerol, nabilone | 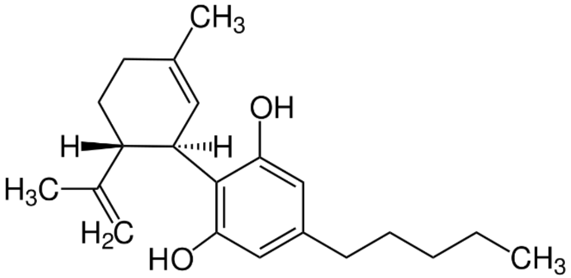  | IOP reduction by 20-30%, maximal effect at 1 hour<br>CB-1 receptors suppress neurotransmitter release at presynaptic neurons (on the ciliary body, ciliary muscles, TM, SC). It affects aqueous humor production and trabecular and uveoscleral outflow<br>CB-2 receptors modulate cytokine release in the immune system<br>Neuroprotective effect by suppressing apoptosis and decreasing free radicals<br>Reduction in reactive oxygen species (ROS), TNF-α and VEGF expression<br>Prevention of superoxide formation, death of retinal cells, vascular hyper-permeability in retinal tissues and reduction of retinal inflammation by inhibition of adenosine uptake in microglia and decreased production of pro-inflammatory cytokines | Psychotropic effects (euphoria, dysphoria, cognitive impairment, decreased short-term memory, time distortion, reduced coordination, somnolence), tachycardia, hypotension, conjunctival hyperemia, corneal injury, local irritation, decreased lacrimation<br>Causes optic nerve ischaemia. Emphysema and lung cancer | Glaucoma | Tomida et al. (2004)<br>Wilkinson & Fraunfelder (2011)<br>Ige & Liu (2020)<br>Pokkalath et al. (2022) |
| ***Carthamus tinctorius* L. (Asteraceae)**<br>TCM: 红花 (Hóng huā)<br>TTM: གུར་གུམ (Gur kum) | **Hydroxysafflor yellow A**, Carthami Flos yellow A, Luteolin, Kaempferol, Adenosine | 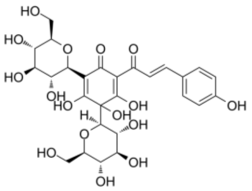 | Can be used to treat early DR and improve the imbalance of VEGF and ES secretion<br>Positive effect on improving serum-related indicators and fundus blood flow in patients with DR<br>Can reduce the contents of VEGF and PDGF in retina<br>Inhibit the formation of new blood vessels and delaying DR | Contraindicated in pregnancy: embryotoxic, increase embryo mortality and intrauterine growth retardation, lead to abortion | DR | Gao et al. (2021) |

| | | | | | | |
|---|---|---|---|---|---|---|
| ***Citrus* spp. L. (Rutaceae),** but can be found in most fruits and vegetables (pineapple, onion, cabbage, tomato etc.) | Vitamin C (**L-ascorbic acid**) and its metabolite (O-methylascorbate) | 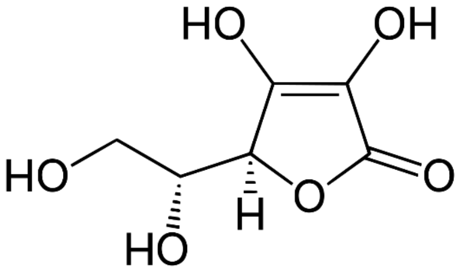 | Dose-dependent high and effective antioxidant capacity against oxidative insults (ROS formation, ion homeostasis, activation of autophagy) in TM cells<br>Induces the viscosity and synthesis of hyaluronic acid and increases aqueous humor outflow through the TM<br>Significant IOP-lowering effect of the metabolite | Generally safe, but in some patient can cause nausea, vomiting, diarrhea, heartburn, stomach cramps or bloating, fatigue and sleepiness, or sometimes insomnia, Headache, skin flushing | Glaucoma<br>AMD | Adornetto et al. (2020)<br>Ikonne et al. (2020) |
| ***Cnidium officinale* Makino (Apiaceae)** | alkylphthalides [Butylidenephthalide (BP)], Cnidilide, Ferulic acid, **Chlorogenic acid**, senkyunolide I, senkyunolide H, senkyunolide A , (Z)-Ligustilide, levistolide A | 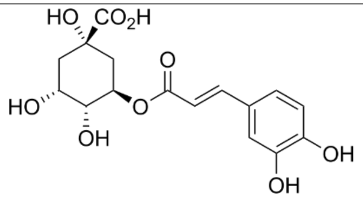 | Anti-angiogenesis: activation of the p38 and ERK1/2 signaling pathways<br>Reduction of neovascular and avascular areas<br>Suppress VEGF expression and angiogenic factors (AREG, ANG, DLL4, IGFBP-2, IL-1α) | No reported toxicity and side effects | DR | Lee et al. (2016)<br>Lim et al. (2019)<br>Tsang et al. (2021) |
| ***Coffea arabica* L. (Rubiaceae)<br>*Coffea canephora* Pierre ex A. Froehner (Rubiaceae)<br>*Ilex paraguariensis* A.St.-Hil. (Aquifoliaceae)<br>*Illicium verum* Hook.f. (Schisandraceae)** | **Caffeic acid**, caffeine (1,3,7-trimethylxanthine), chlorogenic acid | 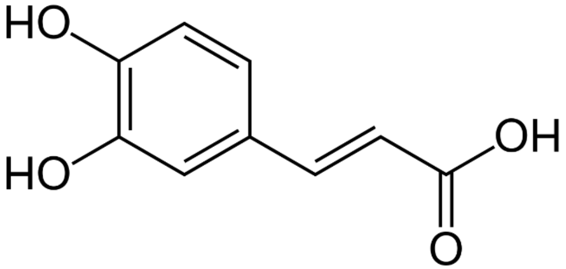 | Reduces ROS, IL-6, TNF-α<br>Suppresses lipid peroxidation and DNA damage<br>Decrease the SOD, CAT, MDA activities<br>Protective in ischemic diseases (suppresses VEGF)<br>In human retina microvascular endothelial cells significantly decreases cell proliferation, cell migration and tube formation (dose-dependent) | Generally safe<br>According to the latest reports, coffee consumption does not affect the IOP<br>Because of the caffeine content, is contraindicated in pregnancy, and can cause minor side effects | DR<br>ROP | Ikonne et al. (2020)<br>Tsang et al. (2021)<br>Pokkalath et al. (2022)<br>Trier et al. (2022) |
| ***Coleus Forskohlii* (Willd.) Briq. (Lamiaceae)** | **Forskolin** | 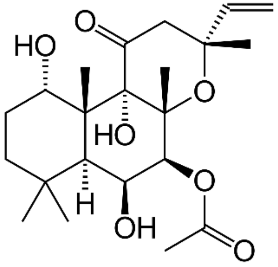 | Activates the catalytic subunit of adenylate cyclase, increasing the intracellular levels of cAMP<br>Reduces IOP<br>Neuroprotective: activation and potentiation of neurotrophins (BDNF, CNTF, IGF-1)<br>Reduces calpain activity and upregulates PI3K/Akt pathway, while it was insensitive to PKA inhibition and independent from the IOP hypotensive action | Can be a safe alternative to beta blockers in glaucoma patients having concomitant asthma | Glaucoma | Adornetto et al. (2020)<br>Pokkalath et al. (2022) |

| | | | | | | |
|---|---|---|---|---|---|---|
| ***Crocus sativus* L. (Iridaceae)** | **Crocin**, Crocetin, Picrocrocin, Safranal, flavonoids (quercetin, kaempferol) | 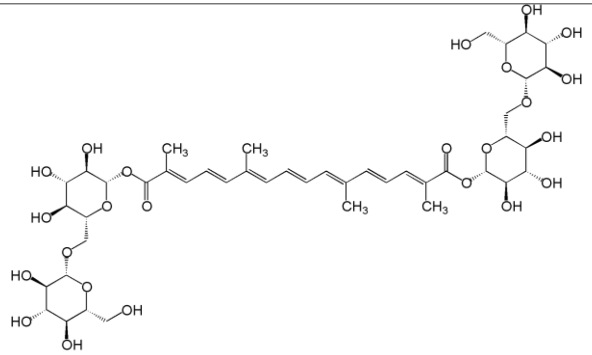 | Inhibits apoptotic pathways: NF-κB, p-ERK, NO, caspase-3, caspase-9, p38, JNK/c-jun, iNOS, MMP3 and cytokins (TNFα, IL-1β, IL-6, CCL2) and ROS from activated microglias and retinal neurons<br>Reduce retinal oedema in CRVO and protection against light-induced retinal damage<br>Raise chorioretinal blood flow, thus improving oxygenation and nutrient supply and facilitating retinal function recovery<br>Activate PI3K/Akt pathway<br>Upregulation of antioxidant enzymes (SOD, GSH)<br>Decrease IOP<br>Reduction of intracellular $Ca^{2+}$-increase induced by ATP acting on purinergic P2X7 receptor<br>Apoptosis prevention: LDH release, intracellular ATP depletion and nuclear condensation<br>More effective than β-carotene to preserve photoreceptor functionality<br>Decrease serum HbA1c and free blood glucose level | Very low toxicity (lethal dose can be above 20g)<br>Dye constituents can accumulate in the skin, mucosas and/or sclera mimicking icteric symptoms<br>Caution in renal insufficiency or bleeding disorders (anti-coagulation treatment)<br>Potential emmenagogue and abortifacient (above 5g), thus it should be avoided in pregnancy | DR<br>Glaucoma<br>iAMD | Heitmar et al. (2019)<br>Adornetto et al. (2020)<br>Camelo et al. (2020)<br>Ige & Liu (2020) |
| ***Curcuma longa* L., (Zingiberaceae)**<br>TCM: 姜黄 (Jiānghuáng)<br>TTM: ཡུང་བ (Yung ba)<br><br>combined with ***Piper nigrum* L., (Piperaceae)** | Essential oils, **Curcumin**, Dihydrocurcumin (DHC), Tetrahydrocurcumin (THC), Hexahydrocurcumin (HHC), Octahydrocurcumin (OHC), Demethoxycurcumin, Bisdemethoxycurcumin, Polyphenols<br><br>Piperine, Sulfur dioxide, Selenium, Vitamin B, Beta-carotene, Curcumin | 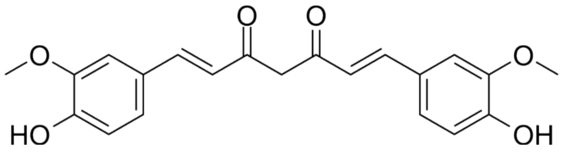 | NSAID-like anti-inflammatory effect (inhibit COX-2 & 5-LOX, iNOS), activate PPAR-γ and BCl-2<br>Induce SOD, Catalase, GSH and GPx expression<br>Antioxidative effect via Nrf2/HO-1 and SIRT1 pathway<br>Reduce astrogliosis by downregulation of JAK2, inhibit NF-κB, Akt, mTOR, MCP-1 and STAT3<br>Reduce pro-inflammatory gene (TIMP1, ICAM1, MMP2, MMP3, CCl2, Fos1, uPA) and cytokine (TNFα, IL-1α, IL-6, IL-8, ELAM-1) expressions in retina and TM<br>Modulate VEGF-VEGR-K-Ras pathway (inhibit HIF-1 and Ets-1)<br>Ameliorate cognitive deficits, neuroinflammation and plaque pathology in Alzheimer's disease, neuroprotection in cerebral ischemia (by glutamate excitotoxicity and NMDA-induced apoptosis)<br>Reduce carbonylated protein levels and decrease the senescence marker SA-β-gal activity | Limited oral absorption and systemic availability<br>Well tolerated and non toxic to animals and humans even at higher doses (e.g. 8-12 g/day) | Glaucoma<br>DR<br>AMD | Alok et al. (2014)<br>Anand et al. (2014)<br>Salehi et al. (2018)<br>Radomska-Leśniewska (2019)<br>Adornetto et al. (2020)<br>Ikonne et al. (2020)<br>Gao et al. (2021)<br>Kao et al. (2021)<br>Tsang et al. (2021)<br>Chiosi et al. (2022)<br>Pokkalath et al. (2022) |

| | | | | | |
|---|---|---|---|---|---|
| | | Inhibit endothelial cell migration and transcription factors (bFGF, FGF-2,IGF-2, TGF-β, TNF, HGF, VEGF)<br>Normalize diabetic microvasculature, attenuates its tortuosity, shrinkage, narrowing and microaneurysms. | | | |
| ***Derris trifoliata*** **Lour. (Leguminosae)**<br>***Mundulea sericea*** **(Willd.) A.Chev. (Fabaceae)** | **Deguelin**<br>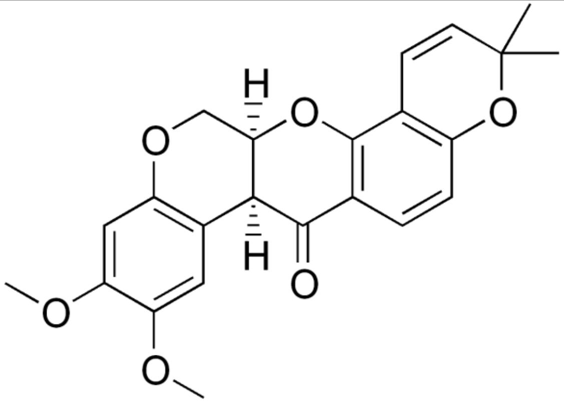 | Inhibiting COX-2 production and downregulating Akt pathway<br>Inhibiting HIF-1α and VEGF expressions therefore reduces NV in the choroid, inhibits tube formation and suppresses vascular leakage | Deguelin is considered a pesticide, and several existing studies have shown that rotenoids are toxic by oral administration (median lethal dose in rats, 132 mg/kg)<br>Associated symptoms of toxicity include respiratory depression, cardiotoxicity, and nerve conduction blockade at high doses (doses that are lethal to 50% of those exposed = 10-100 g in humans)<br>Other routes of administration have also been shown to induce toxic effects (subcutaneous treatment of rats with deguelin has been shown to induce a Parkinson disease like syndrome) | DR | Varughese et al. (2019)<br>Tsang et al. (2021) |
| **Egg yolk, olive oil, carrots, dark green vegetables, but mostly extracted from *Tagetes erecta* L. (Asteraceae)** | Xanthophyll carotenoids (**lutein, zeaxanthin**, meso-zeaxanthin)<br>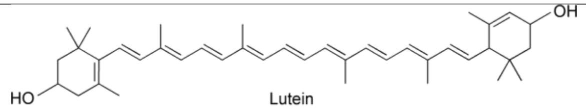<br><br>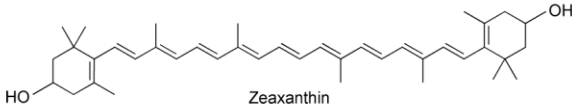<br> | Retinal neuroprotection: maintain cell viability and reduce inflammation after hypoxic damage in RGCs and Müller cells<br>Anti-apoptotic: suppress oxidative stress by inhibiting NF-κB pathway and apoptotic pathway<br>Anti-inflammatory<br>Suppress GFAP immunoreactivity<br>Mainly accumulate in the macula and crystalline lens: prevention of photo-oxidative cataract and AMD<br>Reduce vascular leakage and avascularization<br>Reduce the diabetes induced GSH decrease | No reported toxicity and side effects | AMD<br>DR | Camelo et al. (2020)<br>Ikonne et a. (2020) |

| | | | | | | |
|---|---|---|---|---|---|---|
| ***Eisenia bicyclis* (Kjellman) Setchell (Lessoniaceae)** | Phlorotannins (**eckol**, 6,6′-bieckol, 8,8′-bieckol, dieckol, phlorofucofuroeckol A) and phloroglucinol derivates | 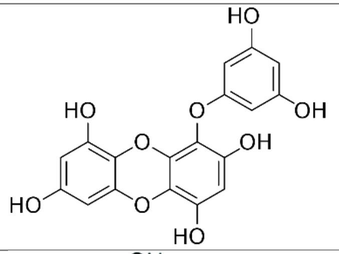 | Protective for RGC cell death caused by ischemia and oxidative stress<br>Scavenge intracellular radical production in RGC cells caused by extracellular $H_2O_2$, $O_2{\cdot}^-$, or ·OH in a concentration dependent manner<br>Decreases glutamate excitotoxicity in a concentration dependent manner<br>Inhibitory effect on β-amyloid precursor protein cleaving enzyme 1 | No reported toxicity and side effects | Glaucoma | Kim et al. (2012)<br>Anand et al. (2014) |
| ***Erigeron breviscapus* (vant.) Hand. Mazz. (Asteraceae)** | Scutellarin, **breviscapine**, erigerone, breviscapinum, flavone glucuronide (5,6,4′-trihydroxyflavone-7-O-glucuronide), 3,5-dicaffeoylquinic acid, erigoster B | 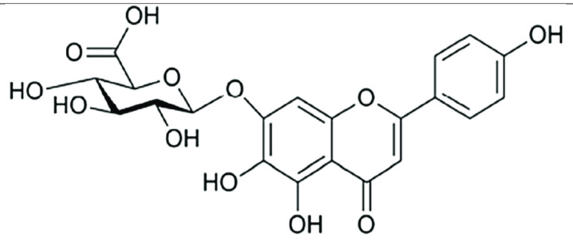 | Tibetan folk medicine applied against headache and eye pain<br>Prevent neuronal apoptosis induced by transient focal ischemia<br>Inhibit the apoptosis of neural stem cells and promoting their differentiation into myelin-producing oligodendrocytes (in Multiple Sclerosis)<br>Improve the ON axoplasmatic transport altered by acute IOP elevation and prevented RGC death induced by ON trauma<br>Visual function improvement by restoring mfERG altered by elevated IOP<br>Inhibit abnormal activation of microglia<br>Protect against hypoxic retinopathy via the inhibition of NLRP3 inflammasome signaling pathway | No reported toxicity and side effects | Glaucoma<br>DR | Adornetto et al. (2020) |
| ***Eriodictyon californicum* (Hook. & Arn.) Torr. (Boraginaceae)** | **Eriodyctiol**, sterubin | 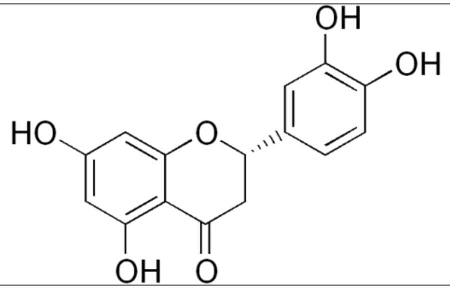 | Reduce TNFα, VEGF, ICAM-1 and eNOS<br>Prevents BRB breakdown | No reported toxicity and side effects | DR | Bucolo et al. (2012) |

| | | | | | | |
|---|---|---|---|---|---|---|
| ***Ginkgo biloba* L. (Ginkgoaceae)**<br>TCM: 银杏 (Yínxìng) | Biflavonoids (ginkgetin, isoginkgetin), terpene trilactones (**ginkgolide**s A, B, C, J, P and Q, and bilobalides), flavonol glycosides (quercetin, kaempherol, isorhamnetin, catechin, myricetin, laricitrin, mearnsetin, apigenin) and proanthocyanidins | 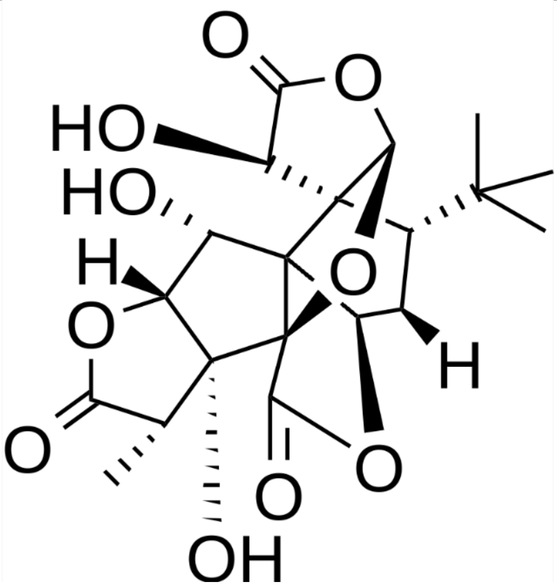 | Downregulate the expression of PAF, therefore preventing leucocyte-endothelial interaction and inflammation<br>Inhibit caspase-activated and anti-Fas ligand-induced apoptosis<br>Reduce the transcriptional expressions of HIF-1α and VEGF<br>Increase peripapillary blood flow, ophthalmic artery velocity, blood viscosity reduction and vasorelaxation<br>Increase photoreceptor cell survival by partially inhibiting the apoptosis of photoreceptor cells<br>Prevent inflammatory reaction, reduced vitreoretinal proliferation, and decreased the frequency of retinal detachment<br>ROS an RNS scavenging: inhibit the protein and lipid oxidation, mitochondria stabilization<br>Effective against dexamethasone induced ocular hypertension, reduce IOP and protect TM<br>Protect neurons against necrosis and apoptosis induced by ROS, Ca2+ overload, NO and βA | Well tolerated and low side effect<br>Bleeding (hyphema and retinal hemorrhages) but no added risk compared with acetylsalicylic acid<br>Low concentration of ginkgolic acids (0.0005%), which is known to cause allergenic and genotoxic effects, but toxic effects in humans are not expected | AMD<br>DR<br>Glaucoma (especially steroid-induced secondary)<br>ROP | Wilkinson & Fraunfelder (2012)<br>Anand et al. (2014)<br>Behl & Kotwani (2017)<br>Kang & Lin (2018)<br>Martínez-Solis et al. (2019)<br>Adornetto et al. (2020)<br>Ige & Liu (2020) |
| ***Lycium barbarum* L. (Solanaceae)**<br>TCM: 枸杞子 (Gǒuqǐ zi)<br>TTM: རདྲེ་ཚོར་མ། (Radre chor ma) | **Lycium barbarum polysaccharides** [(LBP): rhamnose, xylose, glucose, mannose, arabinoside, galactose], zeaxanthin, lutein, carotene, betaine, cerebroside, β-sitosterol, p-coumaric, and various vitamins (riboflavin, thiamin, ascorbic acid) | 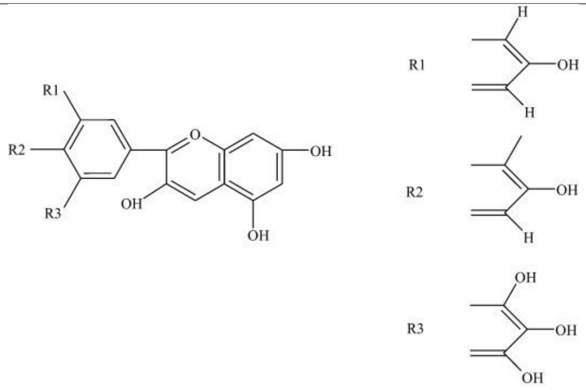 | Upregulate the expression of anti-apoptotic gene Bcl-2<br>Downregulate the expression of pro-apoptotic gene Bax and VEGF mRNA, VEGF-A, VEGF-R2, ANG2, ASM, while increase ANG1 protein expression<br>Inhibit the activation of cyt *c*/caspase-3-mediated apoptotic pathway by blocking $Ca^{2+}$-dependent mitochondrial permeability transition pore<br>Prevent glutamate-mediated excitotoxicity-induced retinal neuronal apoptosis and reduce photoreceptor apoptosis<br>Activates PPAR-γ receptor, Nrf2/HO-1 and JNK/c-jun pathway<br>Maintain the integrity of BRB by increasing P-occludin, inhibiting the Rho/ROCK pathway, and the expression of ICAM-1, P-MLC<br>Anti-aging and immunomodulatory effect: decreasing immune intensity | No significant adverse effects, exception of less minor allergic reactions | DR<br>Glaucoma | Mi et al. (2013)<br>Behl & Kotwani (2017)<br>Manthey et al. (2017)<br>Yang et al. (2020)<br>Gao et al. (2021) |

| | | | | | | |
|---|---|---|---|---|---|---|
| | | | of GFAP and increasing PEDF expression<br>Downregulation of RAGE and their ligands (AGE), ET-1, Aβ<br>Modulation of microglia/macrophage activation (macrophage M2 polarization), MAPK pathway in the inner retina, direct upregulation of neuronal crystallins (βB2-crystallin)<br>Reduction of autophagy marker LC3II, inhibit RPE autophagy<br>Decrease AQP4 and downregulate IgG exosmosis and PAR expression<br>Significantly reduce NLRP3, IL-1β, IL-18 and P62<br>Increase PI3K, P-mTOR/mTOR and P-Akt/Akt levels<br>Significantly increase the serum levels of SOD, GSH, GPx, while decrease serum MDA and creatine<br>Decrease TNF-α, IL-6β, CCL-2, NF-κB in microglias | | | |
| ***Magnolia officinalis* Rehder & E.H.Wilson (Magnoliaceae)**<br>TCM: 厚朴 (Hòu pǔ) | Polyphenol (**Honokiol**) | 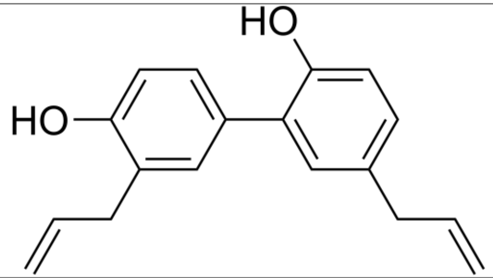 | VEGF receptor antagonist<br>Anti-inflammatory: inhibits NF-κB and its gene products (survivin, Bcl-2, Bcl-xL, IAP1, IAP2, cFLIP and TRAF1), suppress TNFα and IL-8 expressions<br>Inhibits both HIF-1α and VEGF pathway and neovascular tuft formation in RPE<br>Effect on neuronal responses is still unclear | No significant toxicity and side effects to normal cell lines<br>Not recommended in pregnancy | DR | Ong et al. (2020)<br>Tsang et al. (2021) |
| ***Matricaria recutita* L. (Asteraceae)**<br>***Apium graveolens* L. (Apiaceae)**<br>***Capsicum annuum* L. (Solanaceae)**<br>and other fruits and vegetables (parsley, broccoli, onion leaves, carrots, peppers, cabbages, apple skins, chrysanthemum) | **Luteolin** (3′,4′,5,7-tetrahydroxyflavone) | 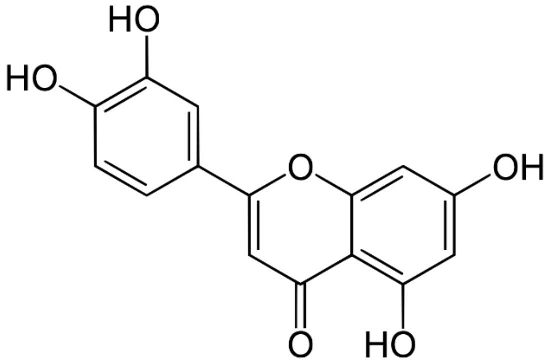 | Permeable to BBB<br>Antioxidant: Modulates ROS and RNS production, enhances endogenous antioxidants (GST, GR, SOD, CAT), suppresses lipoxygenase, cyclooxygenase, and ascorbic acid-stimulated malonaldehyde formation<br>Inhibits the activation of TNFα, ILs<br>Reduce HIF-1α and VEGF expression: affect ROP progression<br>Inhibits the activation of VEGF-R and the downstream pathways: PI3K/Akt and PI3K/p70S6<br>Suppresses NV and migration and tube formation of the retinal endothelial cells<br>Suppresses angiogenesis by stabilizing hyaluronic acid | Generally safe, but in higher doses can cause minor side effects | DR<br>ROP | Lin et al. (2008)<br>Tsang et al. (2021) |

| | | | | | | |
|---|---|---|---|---|---|---|
| | | | Suppresses the NF-κB pathway and MAPK family (ERK, p38, JNK) | | | |
| ***Miyamayomena koraiensis*** **(Nakai) Kitam. (Asteraceae)** | Astersaponin I (AKNS-2), **Chlorogenic acid**, 3,5-di-O-caffeoylquinic acid | 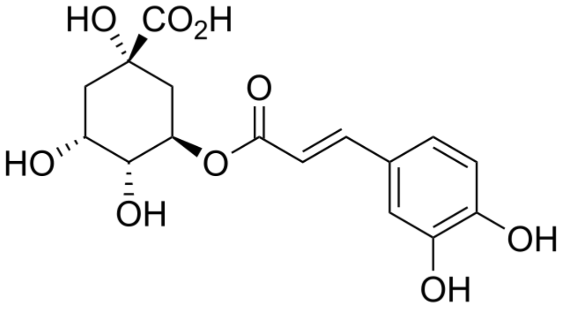 | Anti-angiogenesis<br>Reduction of neovascular area and apoptosis<br>Suppression of VEGF expression (VEGF-α)<br>Attenuates retinal IL-1β, TNF-α, IL-6, IL-8, and MMP-9 in a dose-dependent manner (decreased MAPK pathway)<br>Restore occludin expression in a dose-dependent manner in BRB<br>Dose-dependent reduction in retinal accumulation of AGEs<br>Neuroprotective effect (autophagy-induction by activating Erk and AMPK signaling) | Low side effects and safe for long-term intake | AMD<br>DR | Hong et al. (2020)<br>Tsang et al. (2021)<br>Zhang et al. (2021) |
| ***Morinda citrifolia*** **L. (Rubiaceae)**<br>TCM: 巴戟天 (Bā jǐ tiān) | Oligosaccharides (baijijiasu, 1,5-anhydro-2,3,4,6-tetra-O-methyl-d-glucitol, 2,5-anhydro-1,3,4,6-tetra-O-methyl-d-mannitol arabinose, galacturonic acid, and galactose, rhamnose), anthraquinones (damnacanthal-3-O-β-D-lucidinmprimeveroside and 3-O-β-D-primeveroside), iridoid glycosides (**asperulosidic acid**) | 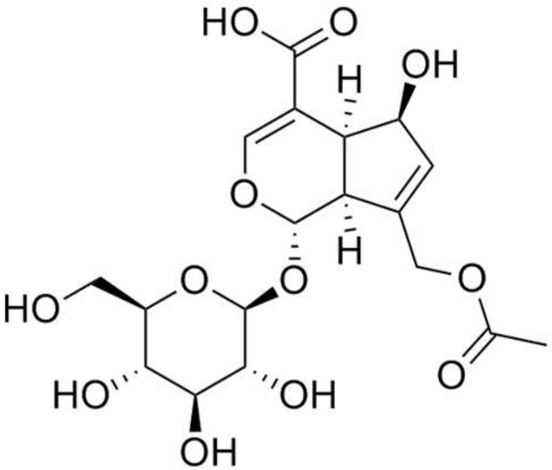 | Can cross the BBB and play beneficial neuroactive roles in various aging and neurodegenerative disease models, including Alzheimer's disease<br>Inhibit TNF-α and IL-1β<br>Reduce the activity of NF-κB<br>Inhibit erythrocyte aggregation and thrombin activity, asperulosidic acid may have clinical applications in improving blood fluidity<br>Inhibit early angiogenesis<br>Significantly reduce serum LPO while enhancing SOD and catalase CAT activities<br>Improvement of the glucose metabolism, via phosphorylation of FOXO1<br>Reduce the number of caspase-3-positive cells, while the memantine had no effect<br>Attenuate oxidative stress and AChE activity | Orally or dermatologically might be safe in appropriate doses<br>Few side effects have been reported<br>Contains a substantial amount of potassium, thus consultation with a healthcare provider is needed<br>Should not be used during pregnancy because the lack of data | DR | Torres et al. (2017)<br>Dheyab et al. (2020)<br>Zhang & Zhang (2022) |

| | | | | | | |
|---|---|---|---|---|---|---|
| ***Panax notoginseng* (Burkill) F.H.Chen (Araliaceae)**<br>TCM: 三七 (Sānqī)<br><br>***Panax quinquefolius* L. *(Araliaceae)*** | Saponins (**Ginsenoside** Re 14, ginsenoside Rd, ginsenoside Rg1, ginsenoside Rb1, notoginsenoside R1), panaxosides, protopanaxatriol derivatives | 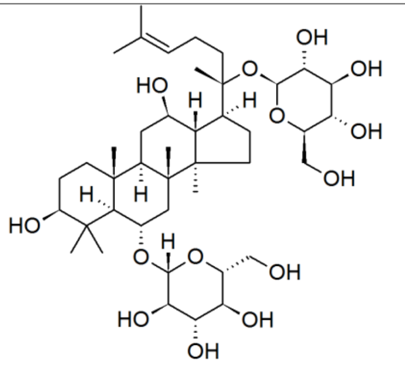 | Prevent ROS-induced apoptosis of the RPE by scavenging hydroxyl and superoxide radicals<br>Downregulation in the mRNA expression of MCP-1 and NF-κB<br>Inhibit VEGF-release from the RPE and AGE-induced retinal endothelial permeability, thereby reducing retina oedema<br>Stabilize the tight junction proteins at the BRB<br>Antidiabetic: lower fasting blood glucose level, improve glucose tolerance and antihyperlipidemic effect | Possibly safe orally or intravenously<br>Can cause dry mouth, flushed skin, rash, nervousness, sleep problems, headache, nausea, and vomiting | DR | Kim & Park (2003)<br>Wang et al. (2008)<br>Behl & Kotwani (2017)<br>Pokkalath et al. (2022) |
| ***Phellodendron chinense* C.K.Schneid.**<br>TCM: 黄柏 (huáng bò) | Alkaloids (Berberine, **Phellodendrine** etc.), Isoquinoline alkaloids (Armepavine, Oxyberberine), Limonoids, Herculin, Phenolic acid, Quinic acid, Hydroxycinnamic acid, Phytosterols, Lignans, Flavonoids (Quercetin, Kaempferol, Phellodendroside, Phellamurin, Phellatin, Phellavin, Phellamuretin, Phelloside etc.), 7-Dehydrostigmasterol, Monosaccharides, Phellolactone, Vanillin, Glycosides | 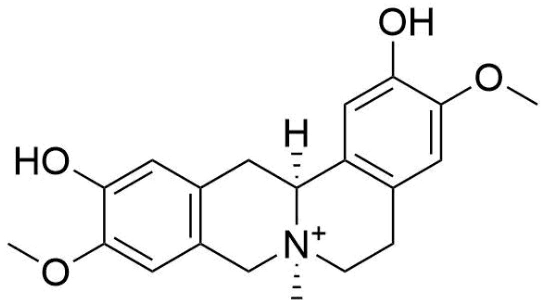 | TMM recommends being included Phellodendri cortex in the herbal formulas treating eye (and ear) diseases<br>Inhibit the release of NO and iNOS production and downregulate NF-κB pathway in microglia<br>Attenuate TNF-α and IL-1β release from microglia<br>Reduce MPO activity by restraining leukocyte mobility and/or secretory activity<br>No effect on PLA2 activity and less effect in AA induced oedema<br>Can play an antioxidant role by modulating the AKT/NF-κB pathway and can inhibit the expression of Akt, NF-κB, IKK, and COX-2<br>Neuroprotective effect: hinder the release of cyt C into the cytosol<br>Can delay or even prevent the progression of diabetes by correcting the high blood sugar state, antioxidant enzyme system, and organ malfunction and reversing histopathological changes inflicted by diabetes | Contraindicated in neonates because can cause neonatal jaundice and kernicterus<br>Besides clinically safe even in patients who have hematological diseases with profound cytopenia and multiple comorbidities. Despite these, bilirubin and hemoglobin monitoring are still required for the patients who have underlining hemolytic disease | DR | Sun et al. (2019)<br>Qu et al. (2022) |

| | | | | | |
|---|---|---|---|---|---|
| ***Pilocarpus jaborandi* Holmes (Rutaceae)** | **Pilocarpine**<br>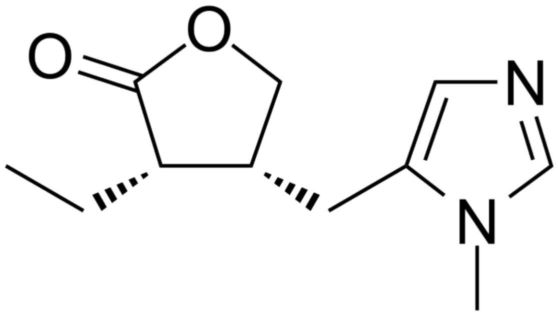<br> | Causes the contraction of the ciliary muscles, which facilitates the aqueous humor outflow through the TM | Local side-effects are a varying degree of annoying involuntary accommodation in patients younger than 45 years and pupillary constriction, which is inconvenient at night and can reduce visual acuity in eyes with cataract, yet increases the depth of focus because of the stenopeic effect<br>Headache and higher risk for tractional retinal detachment in high myopes<br>Do not have major systemic side-effects | Glaucoma | Jonas et al. (2017)<br>Ige & Liu (2020) |
| ***Pinus pinaster* Aiton (Pinaceae)** | Pycnogenol® [organic acids (p-hydroxybenzoic, protocatechuic, gallic, vanillic, p-coumaric, caffeic and ferulic acid), taxifolin, **catechin**, and flavan-3-ols standardized to 70 ± 5% procyanidins]<br>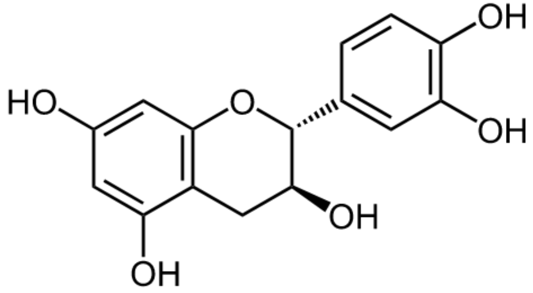<br> | Increases endothelium-dependent vasodilation and decreases systolic and diastolic blood pressure in hypertensive patients<br>IOP reduction hypothetically by the effects on aqueous humor and TM redox status<br>Possess pronounced affinity to collagen, which is believed to be responsible for the reduction of a pathologically increased capillary permeability<br>Dose-dependent stabilization of capillary walls: the effect lasts for up to 8 hours thus superior to O-(β-hydroxyethyl)-rutin and hesperidin methyl-chalcone<br>Antioxidant potential (SOD scavenging)<br>Anti-inflammatory action: reduce leukocyte-mediated degeneration of retinal capillaries<br>Reduces: NF-κB, ICAM-1, VCAM-1<br>Inhibitory on platelet activation | No reported toxicity and side effects | DR<br>Glaucoma | Schönlau & Rohdewald (2001)<br>Karhanová et al. (2015)<br>Manabe et al. (2021) |
| Present in algae (e.g. ***Haematococcus pluvialis, Chlorella zofingiensis and Xanthophyllomyces dendrorhous),*** plants and seafood | **Astaxanthin**<br>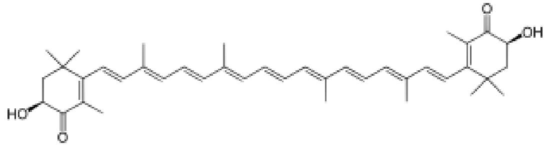<br> | Shows reduced retinal injury (in elevated IOP)<br>Inhibit neurotoxicity induced by ROS<br>Antioxidant: oxidative stress markers (8-OHdG, MDA) are reduced in the serum<br>Oral intake reduced total hydroperoxide in aqueous humor | Possibly safe<br>Might cause increased bowel movements and red stool color | AMD<br>DR<br>Glaucoma | Anand et al. (2014)<br>Giannaccare et al. (2020)<br>Manabe et al. (2021)<br>Pokkalath et al. (2022) |

| | | | | | |
|---|---|---|---|---|---|
| | | Inhibits NF-κB activity, and thereby attenuates RGCs apoptosis<br>Downregulate mTOR complexes (AMPK-mTOR signaling pathway), thus regulating autophagy<br>Able to pass BBB<br>Significant suppression of VEGF, ICAM-1, IL-6, MCP-1, therefore CNV<br>Acts against apoptosis by blocking p-ERk/ERK, cyt C, caspase 3,9 and Bax2/Bcl2 ratio | | | |
| ***Pueraria montana var. lobata* (Willd.) Maesen & S. M. Almeida ex Sanjappa & Predeep (Fabaceae)**<br>TCM: 葛根 (Gégēn) | Isoflavonoids (**Puerarin**, genistein and daidzein)<br>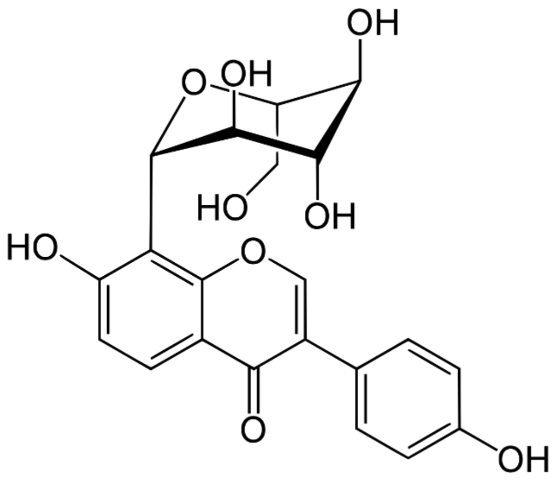 | Prevent peroxynitrite-induced cellular apoptosis (reducing mRNA expression of iNOS and enhancing SOD activity)<br>Attenuate AGE-induced oxidative stress (inhibiting AGE receptor expression)<br>Prevents retinal pericyte apoptosis by suppressing the activation of NADPH oxidase (by inhibiting p47phox and Rac1-mediated signaling), NF-κB, VEGF and HIF-1α<br>Inhibit tyrosine kinase (TNF-α) by inhibiting microglial activation<br>Inhibition of ERK and p38 MAPK phosphorylation<br>Anti-inflammatory effects by PPAR-γ receptor agonism<br>Prevent leucocyte–endothelial interaction (attenuate IL-1β, inhibiting ICAM-1 and VCAM-1), leakage, and oedema<br>Decrease the RPE apoptosis: downregulating the expression of Bax, caspase-3 and upregulating the expression of Bcl-2 which lead prevention of BRB breakdown and vascular stability | Possibly safe by oral consumption<br>Contains ingredients that might counteract the effects of alcohol | DR | Behl & Kotwani (2017)<br>Pokkalath et al. (2022) |
| ***Salvia miltiorrhiza* Bunge (Lamiaceae)**<br>TCM: 丹參 (dānshēn) | **Salvianolic acid A**, rosmarinic acid<br>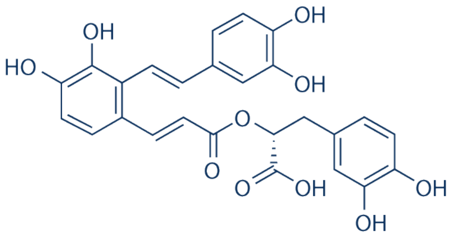 | Upregulate endogenous antioxidant enzymes (catalase, SOD, eNOS, glutathione peroxidase)<br>Inhibits the production of SOD in microglia<br>Inhibit Ang-II-induced NADPH oxidase-4 (Nox4)<br>Preventing endothelial cell proliferation and angiogenesis, improve microcirculation<br>Inhibits retinal NV (anti-VEGF), thickens capillary basement | Possibly safe<br>Not recommended in pregnancy<br>Might slow blood clotting, so there is a concern that it might increase the risk of extra bleeding during and after surgery<br>Interactions with digoxin, midazolam and anti-platelet drugs | DR | Behl & Kotwani (2017)<br>Pokkalath et al. (2022) |

| | | | | | | |
|---|---|---|---|---|---|---|
| | | | membrane, which alters the membrane permeability<br>Inhibit platelet aggregation and promotes fibrinolysis | | | |
| ***Scrophularia ningpoensis* Hemsl. (Scrophulariaceae)**<br>TCM: 玄参 (Xuán shēn) | Angoroside C, acetoside, sibirioside A, 6-O-caffeoyl sucrose, oligosaccharides of raffinose, **harpagoside**, stachyose and verbascose; iridoid glycoside (catalpol, scrophuloside A,B, Ningposide C,D) | 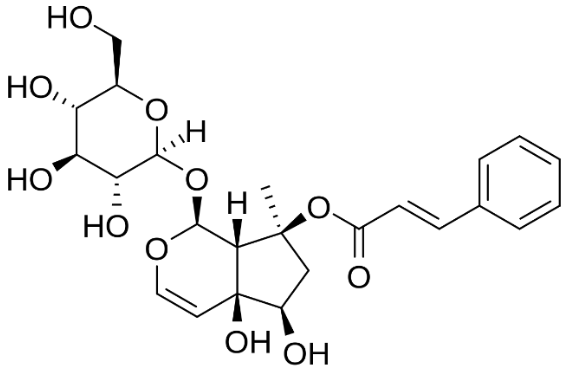 | Aldose reductase inhibitor<br>Downregulate the expression of Nox-4 and its component p22phox<br>Suppress the expressions of various cytokines<br>Inhibit the activity of COX-2, downregulate TNF-α, MCP-1 and NO production and blocking NF-κB pathway<br>Attenuate the mRNA expression of cardiolipin synthetase 1 (which is essential for inflammosome NLRP3 activity) and hinders IL-1β secretion<br>Antioxidant effect by upregulating the activity of SOD | Possibly safe<br>Interaction with Veratrum nigrum causing potential toxicity and a decrease in efficacy<br>Not recommended for patients with spleen deficiency and loose stools | DR | Behl & Kotwani (2017)<br>Ren et al. (2021) |
| ***Scutellaria baicalensis* Georgi (Lamiaceae)**<br>TCM: 黄芩 (huángqín) | **Baicalin**, baicalein, wogonin, norwogonin, oroxylin A, β-sitosterol | 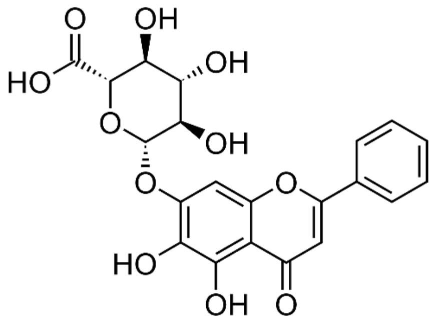 | Anti-oxidative: Reducing iron-accumulation and ROS production, prevent lipid peroxidation<br>Anti-inflammatory: suppressing cytokine production (IL-6, TNF-α)<br>Anti-angiogenic: Inhibits retinal NV (suppressing VEGF, angiotensin II, MMP-9 expression)<br>Reduces vascular abnormality and ganglion cell loss within the retina | Possibly safe<br>It might cause stomach pain, constipation, vomiting, and stomach upset | DR<br>ROP | Tsang et al. (2021)<br>Pokkalath et al. (2022) |
| ***Solanum lycopersicum* L. (Solanaceae),** and watermelon, pink grapefruit, and papaya | **Lycopene** | 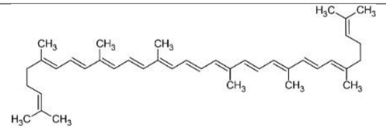 | Anti-inflammatory and anti-oxidative on eye tissues, especially on RPE<br>Improve GSH and MDA activities<br>Neuroprotective<br>Prevents angiogenesis | No reported toxicity and side effects | AMD<br>DR | Ikonne et al. (2020) |
| ***Tinospora cordifolia* (Thunb.) Miers (Menispermaceae)** | **Octacosanol** | 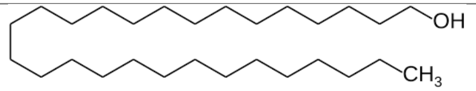 | Downregulate VEGF gene expression by inhibiting MMPs and nuclear translocation of NFκB and reduce its DNA binding activity<br>Increase both glutathione and catalase levels | Reported acute hepatitis in case of overdosage, but other toxicological factors cannot be excluded | DR | Agrawal et al. (2012) |

| | | | | | | |
|---|---|---|---|---|---|---|
| ***Trigonella foenum-graecum* L. (Fabaceae)** | 4-hydroxyisoleucine, protodioscin, **trigonelline**, polyphenols (choline, luteolin, orientin, quercetin) | 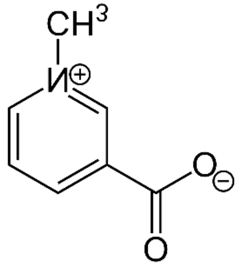 | Inhibits the expression of anti-inflammatory (TNF-α, IL-1β) and anti-angiogenic biomarkers (VEGF, PKC-β)<br>Neuroprotection in Parkinson's Disease | Recognized as safe<br>Ophthalmoscopic observations on animals did not reveal any treatment related side effects (e.g. corneal ulcer, angiogenesis, compression of retinal vessels, glaucoma or intraocular inflammatory changes) | DR | Sureshkumar et al. (2018)<br>Pokkalath et al. (2022) |
| ***Vaccinium myrtillus* L. (Ericaceae)** | Flavonoids (**anthocyanin**s)<br>Mirtoselect® (standardized to contain 36% anthocyanins) | 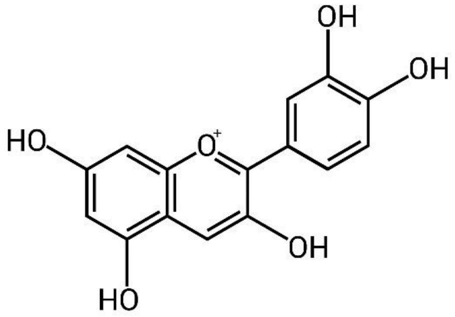 | Prevention of STAT3 activation thereby reducing inflammation-related rhodopsin damage<br>Antioxidative property ameliorating ROS-damage to the retina<br>Anti-apoptotic effect<br>Capillary- and collagen stabilization – protective against BRB breakdown<br>Inhibit lipid peroxidation and proinflammatory cytokines to prevent cell apoptosis in light-induced retinal degeneration<br>Reduction of cell proliferation and inhibition of VEGF-induced phosphorilation of ERK<br>Suppression of neovascular tufts formation<br>Stabilizing optic nerve structure, enhancing the resistance of RGCs to the mechanical and ischemic alteration<br>Decrease neuroinflammation<br>No effect on IOP<br>Improve several retinal microcirculatory and perfusion parameters | No reported toxicity<br>Severe overdosing can cause cachexia, anemia, or icterus | early AMD, Glaucoma, ROP | Anand et al. (2014)<br>Karhanová et al. (2015)<br>Ige & Liu (2020)<br>Manabe et al. (2021)<br>Tsang et al. (2021) |
| ***Vitis* spp. L. (Vitaceae), *Veratrum grandiflorum* O. Loes (Melanthiaceae), *Fallopia japonica* Houtt. (Ronse Decr.) (Polygonaceae)** and blueberries, cranberries, peanuts dark chocolate and red and white wines | Polyphenol (**Resveratrol** = 3,5,4′-trihydroxystilbene) | 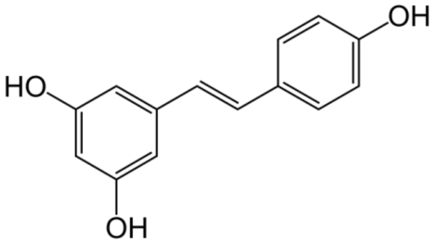 | Represses diabetes-induced oxidative stress, pro-inflammatory cytokines expression, retinal vascular permeability, caspase-3 activity, and apoptosis of the retina, attenuating the retinal inflammatory condition and damage of DR via recovery of expression of PON1<br>Effectively reduce ROS generation and maintain intracellular antioxidant concentration<br>Downregulates iNOS, eNOS and nNOS expressions, and inhibits Bcl-2 and PGE2 receptor expression | Does not appear to have side effects at short-term doses (1.0 g)<br>Otherwise, at daily doses of 2.5 g <, side effects may occur (nausea, vomiting, diarrhea and liver dysfunction in patients with non-alcoholic fatty liver disease) | DR<br>Glaucoma<br>ROP | Salehi et al. (2018)<br>Adornetto et al. (2020)<br>Ikonne et al. (2020)<br>Tsang et al. (2021)<br>Pokkalath et al. (2022) |

| | | | |
|---|---|---|---|
| | | Mediates VEGF expression and suppresses neovascular tuft formation and reduces NV<br>Reduces RGC loss and preserves pupillary light response following ON injury<br>SIRT-1 and AMPK/PGC-1α activation, BiP, CHOP, XBP-1 expression in RGC neuroprotection<br>Preserves retinal thickness and cellular density in GCL by the downregulation of caspase-3 and caspase-8 expression and suppression of gliosis-related inflammation<br>Decreases ROS expression of acetyl-p53, and upregulation BDNF and its (TrkB) receptor<br>In glaucomatous human TM prevents the increase of IL-1α, IL-6, IL-8, ROS, ELAM-1 and reduces the expression of the senescence markers (SA-β-gal, lipofuscin), and the accumulation of carbonylated proteins | |

Color coding: Green: agents take action on antiapoptotic and anti-aging mechanisms. Orange: agents take action on anti-vessel formation mechanisms (anti-VEGF, anti-HIF1α). Yellow: agents take action on anti-inflammatory mechanisms. Blue: agents take action on anti-inflammatory and neuroprotective pathways. Pink: agents take action all mechanisms. Abbreviations: 5-LOX = 5-lipoxigenase, 8-OHdG = 8-hydroxy-2' –deoxyguanosine, A2E = Bis-retinoid N-retinyl-N-retinylidene ethanolamine, AChE = acetylcholinesterase, AGE = advanced glycation endproducts, Akt = PKB = Protein kinase B, AMD = Age-related macular degeneration, AMPK = AMP-activated protein kinase, Ang-II = Angiotensin-II, ATP = adenosine triphosphate, Aβ = amyloid β plaque, Bax = Bcl-2-associated X protein, BBB = blood-brain barrier, Bcl-2 = B-cell lymphoma 2, BDNF = brain-derived neurotrophic factor, bFGF = basic fibroblast growth factor, BiP = Binding immunoglobulin protein, BRB = blood-retinal barrier, CatG = Cathepsin G, CB(-1,2) = cannabionoid-receptor (type1,2), CCl2 = CC motif chemokine ligand 2, CDK9 = Cyclin-dependent kinase 9, CHOP = C/EBP Homologous Protein, CNTF = Ciliary neurotrophic factor, COX-2 = cyclooxigenase-2, CREB = cAMP response element-binding protein, CRVO = central retinal vein occlusion, cyt C = cytochrome C, CSF = cerebrospinal fluid, DR = Diabetic retinopathy, ELAM-1 = endothelial-leukocyte adhesion molecule 1, eNOS = endothelial NOS, ERG = electrogretinography, ERK = extracellular signal-regulated kinase, ET-1 = endothelin-1, FGF = fibroblast growth factor, FOXO-1 = Forkhead box protein O1, GCL = ganglion cell layer, GDNF = glial cell-derived neurotrophic factor, GLUT-4 = Glucose transporter type 4, GSH = glutathione, HbA1c = glycated hemoglobin (hemoglobin A1c), HGF = hepatocyte growth factor, HIF-1α = Hypoxia-inducible factor 1-alpha, HO-1 = heme oxygenase 1, iAMD = intermediate age-related macular degeneration, ICAM-1 = Intercellular Adhesion Molecule 1, Ig = immunoglobulin, IKK = IkappaB kinase, IL = interleukin, iNOS = inducible nitric oxide synthase, IOP = intraocular pressure, JNK = c-Jun N-terminal kinase, KDR/flk-1 = Kinase Insert Domain Receptor/VEGF Receptor-2, LC3II = Microtubule-associated protein light chain 3, LDH = Lactate dehydrogenase, LPO = lipid peroxidation, LPS = lipopolysaccharide, MAPK = mitogen-activated protein kinase, MBP = Maltose-binding protein, MCP-1 = Monocyte Chemoattractant Protein-1, MDA = Malondialdehyde, mfERG = Multifocal electroretinogram, mPGES-1 = Microsomal prostaglandin E synthase-1, MPO = myeloperoxydase, mRNA = messenger ribonucleic acid, NADPH = Nicotinamide adenine dinucleotide phosphate, NF-κB = nuclear factor kappa B, nNOS = neuronal NOS, NO = nitric oxide, Nrf-2 = nuclear factor erythroid 2–related factor 2, NSAID = Nonsteroidal Anti-Inflammatory Drug, NV = neovascularization, OHT = Ocular hypertension, ON = optic nerve, PAF = platelet activating factor, PAI-1 = Plasminogen activator inhibitor-1, PAK1 = Serine/threonine-protein kinase PAK 1, PGC-1α = Peroxisome proliferator-activated receptor gamma coactivator-1 alpha, PGE2 = Prostaglandin E2, PI3K = Phosphoinositide 3-kinase, PKC = Protein kinase C, PON-1 = paraoxonase and arylesterase 1, PPAR-γ = Peroxisome proliferator-activated receptor gamma, RAGE = Receptor for Advanced Glycation Endproducts, RGCs = retinal ganglion cells, ROP = retinopathy of prematurity, ROS = reactive oxygen species, RPE = retinal pigment epithelium, SA-β-gal = Senescence-associated beta-galactosidase, SC = Schlemm's canal, SHP-1 = Src homology region 2 domain-containing phosphatase-1, SIRT1 = Sirtuin-1, SOD = superoxide dismutase, STAT3 = Signal transducer and activator of transcription 3, TCM = Traditional Chinese Medicine, TFs = transcription factors, TGF-β = Transforming Growth Factor-beta, TIMP1 = TIMP metallopeptidase inhibitor 1, TLR = toll-like receptor, TM = trabecular meshwork, TMM = Traditional Mongolian Medicine, TNF-α = tumor necrosis factor alpha, TrkB = tyrosine receptor kinase B, TTM = traditional Tibetan Medicine, uPA = Urinary-type Plasminogen Activator, VCAM-1 = Vascular cell adhesion protein 1, VEGF(-R) = vascular endothelial growth factor (receptor), VPO-1 = Vascular peroxidase 1, XBP1 = X-Box Binding Protein 1.
