# Supplementary material for: Three Major Causes of Metabolic Retinal Degenerations and Three Ways to Avoid Them
Source: Int J Mol Sci. 2023 May 13;24(10):8728. doi: 10.3390/ijms24108728 (PMC10218427; doi:10.3390/ijms24108728)
Supplement: Supplementary file 1 [file ijms-24-08728-s001.zip › TableS2.pdf]

Active ingredient	Pharmacobiologic properties	Structure of main marker molecules	Adverse effects, limitations	Route of application	Target retinal diseases	References
**Acetazolamide sodium**	Carbonic anhydrase inhibitor (CAI)<br>Sulfonamide-derivate	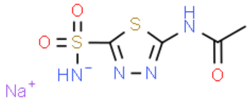	Paresthesia of the acra, fatigue, depression, renal stones, and gastrointestinal complaints such as nausea and diarrhoea	Oral	Glaucoma	Mincione et al. (2008)<br>Farzam et al. (2022)<br>Hoyng et al. (2000)
**Acetyl salicylic acid (Aspirin)**	Anti-inflammatory: non-selective COX inhibitor: irreversibly inhibits COX-1 and COX-2 to decrease conversion of arachidonic acid to precursors of prostaglandins and thromboxanes<br>Inhibit NF-κB	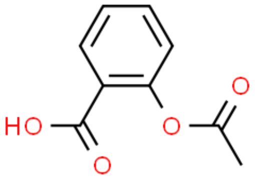	Bleeding risk, inhibits platelet-aggregation	Oral	AMD<br>DR	Zheng et al. (2007)<br>Al-Zamil et al. (2017)
**Aflibercept (Eylea®)**	VEGF inhibitor<br>Dimeric glycoprotein<br>Suppress neovascularization and decrease vascular permeability	$C_{4318}H_{6788}N_{1164}O_{1304}S_{32}$	Increased risk of stroke and myocardial infarction<br>Increase of IOP	Intravitreal	AMD<br>DR	Duh et al. (2017)
**Apraclonidine hydrochloride*** (also known as iopidine)	α2-adrenergic agonist<br>For prevention or reduction of IOP: reducing aqueous humor production and increasing uveoscleral outflow	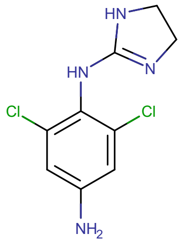	Allergic reactions including eyelid dermatitis, blepharoconjunctivitis and follicular conjunctivitis, hyperemia, itching, tearing, and occasionally foreign body sensation<br>Dry nose and dry mouth<br>Headache, fatigue, and sedation	Topical	DR<br>Glaucoma (a short-term adjunctive therapy)	Koc et al. (2006)<br>Hoyng et al. (2000)
**Betaxolol hydrochloride 0.25%**	Competitive, β1-selective (cardioselective) adrenergic antagonist<br>Less effective than brimonidine 0.2%<br>Reduces IOP<br>Can be used in combination	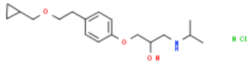	Blurred vision<br>Burning and stinging after topical administration<br>Has less effect on cardiac and pulmonary function than non-selective β-blockers<br>May induce adverse effects on the CNS such as depression, fatigue, impotence, and confusion<br>Special attention in patients with asthma	Topical	Glaucoma	Adkins et al. (1998)<br>Lesar (1987)

**Bevacizumab**	Recombinant monoclonal antibody<br>Binds and inhibits all isoforms of VEGF, but with a lower affinity, and has a longer half-life than Ranibizumab<br>Decreases central retinal thickness	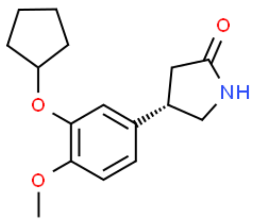	Corneal abrasion, lens injury, endophthalmitis, and retinal detachment<br>Inflammation or uveitis, cataract progression, acute visual loss, CRAO, subretinal hemorrhages, and RPE tears<br>Mild increases in blood pressure, transient ischemic attack, cerebrovascular accident, and death	Intravitreal	AMD<br>DR	Iu et al (2007)<br>Vaziri et al. (2015)<br>Duh et al. (2017)
**Bimatoprost 0.03%**	Prostamide<br>Prostaglandin F2α analogue<br>Reduction of IOP: mildly stimulates aqueous humor outflow via the TM and uveoscleral pathways<br>Significantly more effective than latanoprost	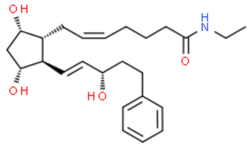	Eye irritation, dry eye, itching, blurred vision, burning, discharge, allergy, and blepharitis,<br>Eyelid pigmentation, changes in iris pigmentation, changes in eyelash pigmentation, growth, and thickness	Topical	Glaucoma	Lim et al (2008)<br>Brubaker (2001)<br>Easthope et al. (2002)<br>Woodward et al. (2008)<br>Alm (2014
**Brimonidine tartrate 0.2%**	Highly selective α2-adrenoceptor agonist<br>Reduces IOP via the uveoscleral pathway: reduction in aqueous humor production and an increase in aqueous humor outflow metabolized in the cornea<br>Undergoes extensive hepatic metabolism, mainly by liver aldehyde oxidase to produce oxo- and dioxo-brimonidine derivatives<br>urinary excretion was the major route of elimination<br>vasoconstrictive effects<br>Activate the intracellular kinases that enhance cell survival, indicate the anti-apoptotic genes such as BCL-2, or neuronal survival factors such as bFGF and inhibit the glutamate release and calcium influx into cells.<br>In vivo mouse inflammation models displayed anti-inflammatory properties by inhibiting edema	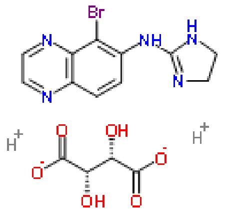	Ocular allergy: eye lid dermatitis, blepharoconjunctivitis and follicular conjunctivitis<br>Blurred vision<br>Decreasing blood pressure, decreasing heart and respiratory rate, and prolonging the PR interval in the electrocardiogram<br><br>Should be used with caution in individuals with severe cardiovascular disease, hepatic or renal impairment, depression, cerebral or coronary insufficiency, Raynaud's phenomenon, orthostatic hypotension or thrombangiitis obliterans antagonists<br><br>Not be used in patients receiving monoamine oxidase inhibitors	Topical (0.08, 0.2 and 0.5% twice daily) or implant	AMD<br>Glaucoma	Adkins et al. (1998)<br>Jackson et al.(2015)<br>Al-Zamil (2017)<br>Weeler et al. (1999, 2001)
**Brinzolamide**	Highly specific, non-competitive, reversible carbonic anhydrase isoenzyme II inhibitor<br>Decreases aqueous humor secretion by slowing the formation of bicarbonate ions with subsequent reduction in sodium and fluid transport<br>Used in combinations	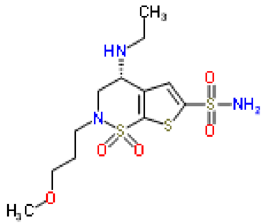	Stinging and burning after instillation (1.8-3%), foreign body sensation (1.8%), itching (1.2%), tearing (1.2%) and dry eyes (1.2%)<br>Taste abnormalities (7.7%)	Topical	Glaucoma	Hoyng et al. (2000)
**Brolucizumab (Beovu®)**	Human antibody fragment<br>Capable of neutralizing all forms of VEGF-A	Not Available	Blurred vision, hazy vision, increased sensitivity to light<br>Eye pain or seeing floaters.	Intravitreal	AMD	Dugel et al. 2017

			Should not use in case of intraocular inflammation or infection<br>Do not use in pregnancy			Motevassali et al . (2021)
**Carbachol 0.75, 1.5 and 3%**	Directly stimulates the muscarinic receptor site<br>Also has an indirect effect by inhibiting cholinesterase<br>Increase TM outflow through ciliary muscle contraction	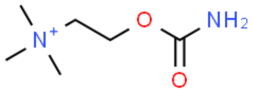	Similar but more serious than pilocarpine	Topical	Glaucoma	Hoyng et al. (2000)
**Carteolol 1 and 2%**	A non-selective β-adrenergic antagonist<br>Decreased aqueous humor production<br>Used in combination	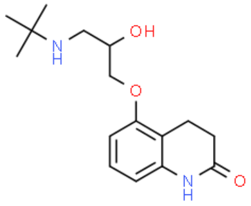	Little or no effect on pupil size<br>Irritation and pain shortly after application<br>Should not be given to patients with cardiac or pulmonary insufficiency	Topical or oral	Glaucoma	El-Kamel et al. (2006)<br>Hoyng et al. (2000)
**Clonidine**	α2 adrenergic agonist<br>Treat hypertension<br>Imidazole derivate<br>Easily passes the BBB, which may result in systemic hypotension by stimulation of the vasomotor centers in the brainstem	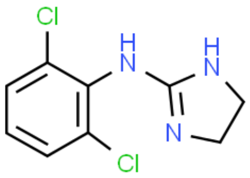	Allergic reactions including eyelid dermatitis, blepharoconjunctivitis and follicular conjunctivitis<br>Hyperaemia, itching, tearing, and occasionally foreign body sensation<br>Dry nose and dry mouth<br>Headache, fatigue and sedation	Topical	Glaucoma	Hoyng et al. (2000)
**Conbercept (Lumitin®, 0.5mg)**	Recombinant fusion protein with high affinity to all VEGF isoforms and PIGF<br>Significantly reduced the CRT	Not Available	Only approved in China<br>Efficacy and safety not yet elucidated in other racial populations	Intravitreal	AMD<br>DR	Zhang et al. (2018)
**Cyclosporine**	Calcineurin inhibitor<br>Immunomodulatory agent<br>Inhibiting the production of cytokines (mainly IL-2) involved in the regulation of T cell activation.<br>Suppressed the production of MHC class 2 (I-a) antigen - inducing lymphokines thereby inhibiting intraocular inflammation<br>Attenuation of retinal expression of inflammatory mediators (IL-1 and TGF-β] and reduced edema and disorganization of the retinal layers	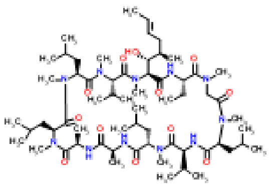	200 µg showed adverse histologic changes in the retina in the form of patchy loss of outer segments of the retina.	Oral,<br>Topical,<br>Intravitreal	DR	Hasan et al. (2022)<br>Wang et al.<br>Zong et al.

**Dexamethasone (Ozurdex®, 700 µg)**	Corticosteroid implant<br>Improve central foveal thickness	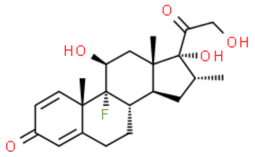	Cataract formation	Intravitreal	DR	Boyer et al. (2014)
**Diclofenamide**	Carbonic anhydrase inhibitor (CAI)<br>Reduces IOP<br>Suppressing the secretion of aqueous humor	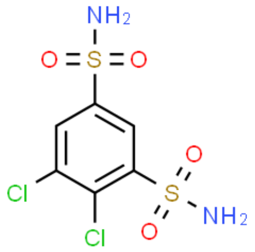	Numbness/tingling, change in the sense of taste, nausea, diarrhea, weight loss, muscle spasms/twitching, tiredness, dizziness, or drowsiness may occur	Oral	Glaucoma	Mincione et al. (2008)
**Dipivefrin hydrochloride**	Phenol esters<br>Prodrug of epinephrine<br>Reduces IOP<br>Stimulating α- and/or β2-adrenergic receptors<br>More lipophilic than epinephrine and penetrates the cornea 17 times more easily	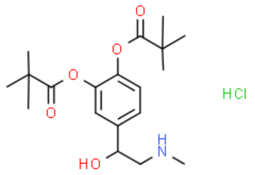	Little or no pharmacologically activity until it is hydrolyzed into epinephrine inside the human eye	Topical	Glaucoma	Arthur et al (2011)
**Dorzolamide hydrochloride 2%**	Carbonic anhydrase isoenzyme II inhibitor in the ciliary process that regulates ion balance and fluid pressure in the eyes<br>Non-bacteriostatic sulfonamide derivative<br>Reduces IOP	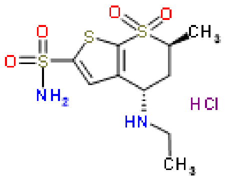	Stinging (12%), ocular burning (19%), temporarily blurred vision (9%), itching (12%) and tearing (7%), eyelid oedema and conjunctivitis (4 and 4.5%)<br>Electrolyte imbalance, acidosis	Topical	Glaucoma	Martens-Lobenhoffer et al. (2002)<br>Balfour et al. (1997)<br>Loftsson et al. (2012)
**Echothiophate iodide**	Long-acting irreversible acetylcholinesterase inhibitor<br>Used in combination	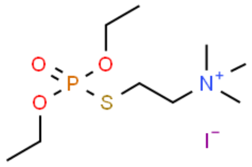	Blurred vision or change in near or distant vision and eye pain	Topical	Glaucoma	Schmidt et al. (2010)

**Fasudil**	Specific ROCK inhibitor<br>Reducing neutrophil-induced endothelial injury<br>significantly increased eNOS phosphorylation<br>Used in combination	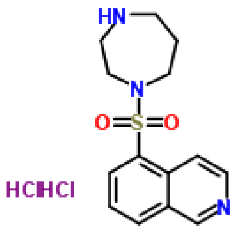	Systemic vasodilation and hypotension	Intravitreal	DR<br>Glaucoma	Rotschild et al. (2005)
**Guanethidine monosulfate**	Postganglionic sympathetic nerve terminal blocker<br>Prevents the release of norepinephrine from nerve terminals<br>Suppresses equally the responses mediated by α-and β-adrenergic receptors but does not produce parasympathetic blockade<br>Guanethidine is converted by the liver to three metabolites, which are excreted in the urine	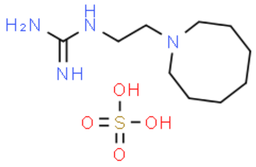	Drowsiness, dizziness, tiredness, or confusion	Oral	Glaucoma	
**Infliximab**	TNF-α Inhibitor<br>Chimeric monoclonal IgG1 antibody<br>Disrupts the proinflammatory cascade signaling so downregulate<br>Proinflammatory cytokines, such as IL-1, IL6 etc.<br>Reduction of lymphocyte and leukocyte migration, apoptosis of TNF producing cells (activated monocytes), and reduction of endothelial adhesion molecules and acute phase proteins.	$C_{6428}H_{9912}N_{1694}O_{1987}S_{46}$	Can be retinotoxic: decline in amplitude and increase latency<br>anterior uveitis	Intravitreal	AMD	Theodossiadis et al. (2009)
**Latanoprost 0.005%**	Isopropyl ester prodrug<br>Analogue of prostaglandin F2α, PhXA34<br>Decreases IOP by increasing uveoscleral outflow	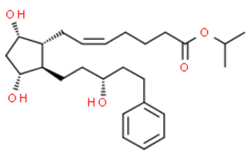	Lower systemic adverse effects than timolol<br>Stinging, burning, or tearing after application<br>Punctate keratitis, blurred vision, eye pain and foreign body sensations<br>Iris pigmentation<br>Infrequent conjunctival hyperemia, pigmentation of periocular tissues, eyelash changes, hypertrichosis, and ocular irritation<br>Should be applied preferably in the evening	Topical	Glaucoma	Alm (2014)<br>Sjoquist et al. (2002)
**Levobunolol hydrochloride 0.5%, 0.25%**	Nonselective β-adrenergic blocking agents: equally effective at β(1)- and β(2)-receptor sites<br>Longer acting than timolol	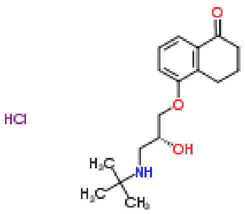	Systemic pulmonary and cardiovascular effects	Topical	Glaucoma	Gonzalez et al. (1987)<br>Lesar (1987)<br>Novack (1986)<br>Ishibashi et al. (2003)

**Memantine**	NMDA receptor blocker<br>Prevents RGC loss<br>Reduces elevated VEGF protein levels<br>Improved amplitudes of ERG a- and b-waves	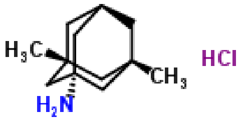	No known adverse effect	Oral	DR<br>Glaucoma	Kusari et al (2007)
**Methazolamide**	Carbonic anhydrase inhibitor<br>Sulfonamide derivative<br>The reduction of elevated IOP in patients who are insufficiently responsive to β-blockers	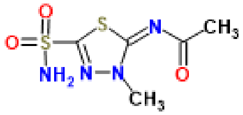	Kidney stones, signs of infection, easy bleeding/bruising, numbness or tingling of hands/feet, tinnitus	Oral	Glaucoma	Skorobohach et al. (2003)
**Metipranolol hydrochloride 0.1, 0.3 and 0.6%**	Non-selective β-adrenergic antagonist<br>the reduction of elevated IOP by inhibiting aqueous humour formation<br>Used in combination	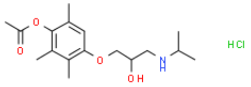	Little or no effect on pupil size<br>Corneal anaesthetic effect<br>Mild conjunctival hyperaemia<br>Slight reduction in pulse rate and systolic blood pressure<br>Plasma HDL, cholesterol levels are increased, and plasma triglyceride levels are reduced<br>Should be avoided in patients with cardiovascular or pulmonary diseases	Topical	Glaucoma	Hoyng et al. (2000)
**Nepafenac (Nevanac ®)**	Nonsteroidal anti-inflammatory drug that inhibits COX-1 and COX-2 and the synthesis of proinflammatory prostaglandins<br>Inhibits caspase 3 and caspase 6	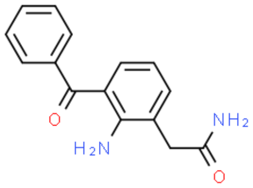	Blurred vision, eye pain or itching, foreign body sensation, sticky sensation of the eyelids	Topical	DR	Kern et al. (2010)
**Netarsudil dihydrochloride 0.02%**	Rho kinase inhibitor with norepinephrine transport inhibitory activity<br>Reduction of IOP	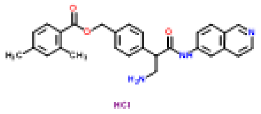	Conjunctival hyperemia, corneal verticillata, instillation site pain and erythema, and even conjunctival hemorrhage, corneal staining, blurred vision, increased lacrimation, erythema of eyelid, and reduced visual acuity	Topical	Glaucoma	Lin et al.(2017)<br>Ren et al. (2016)

**Pegaptanib (Macugen®, 0.3 mg)**	28-base RNA aptamer that selectively binds to and blocks the activity of VEGF	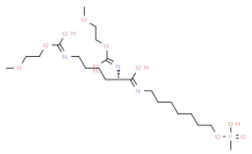	Eye pain, vitreous floaters, keratitis<br>rare (0.1%): endophthalmitis, traumatic injury to the lens, and retinal detachment	Intravitreal	AMD	Vavvas et al. (2006)
**Pilocarpine hydrochloride 4%, 2%, 1%**	Muscarinic cholinergic agonist<br>Produces contraction of the iris sphincter muscle and ciliary muscle: causes miosis, spasm of accommodation<br>Reduces the outflow resistance of aqueous humor through the TM and Schlemm's canal<br>Used in combinations	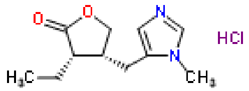	Blurred vision especially in younger patients, causing myopia by for-ward displacement and thickening of the lens<br>Conjunctival hyperemia, lens opacities and retinal detachments<br>paradoxical effects on the cardiovascular system: Bradycardia and tachycardia<br>Vomiting, nausea, diarrhea, bronchospasm, and sweating	Topical	Glaucoma	Adkins et al. (1998)
**Ranibizumab (3 mg and 0.5 mg)**	Recombinant humanized monoclonal antibody and VEGF-A antagonist<br>Inhibits the formation of new blood vessels or neovascularization<br>also reduce retinal thickness	$C_{2158}H_{3282}N_{562}O_{681}S_{12}$	Endophthalmitis, retinal detachment, and traumatic cataract	Intravitreal	AMD<br>DR	Gaudreault et al. (2007)<br>Kourlas et al. (2007)<br>Akiyode et al. (2016)<br>Al-Zamil et al. (2017)<br>Duh et al. (2017)
**Ripasudil hydrochloride dihydrate 0.4%**	Rho kinase inhibitor<br>Hydrochloride hydrate (K-115)<br>Decreasing IOP in a dose-dependent manner and increasing flow facility<br>Inducing cytoskeletal change<br>Used in combination with prostaglandin analogues	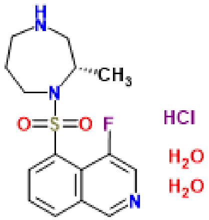	Mild to moderately severe conjunctival hyperemia<br>Mild conjunctival follicles<br>Ocular irritation, abnormal sensation in the eye<br>Conjunctival hemorrhage	Topical	DR<br>Glaucoma	Kaneko et al. (2016)
**Sirolimus (Rapamune)**	Inhibiting T-lymphocyte activation and proliferation stimulated by antigens and cytokines such as IL-2, IL-4, and IL-15<br>Binds to the cytoplasmic receptor FKBP12, an immunophilin, to form an immunosuppressive complex<br>FKBP12-sirolimus complex binds to and inhibits the activation of the mTOR (serine/threonine-specific protein kinase - cell growth, proliferation, survival, mobility, and angiogenesis)<br>mTOR regulates the downstream signaling pathways involved in cell survival, such as	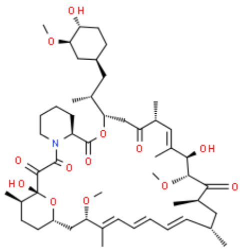	No known adverse effect	Oral	AMD	Hasan et al. (2022)

	the phosphatidylinositol-3 kinase (PI3K)/Akt signaling pathway					
**Tafluprost**	Ester prodrug<br>Prostaglandin analogue high affinity for receptor PGF2<br>Reducing elevated IOP by increasing the outflow of aqueous humor	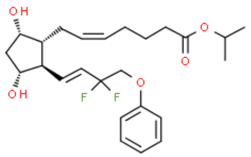	Eye pain or redness, itchy or watery eyes<br>Increased sensitivity to light or severe redness or burning of the eyes after using the drops	Topical	Glaucoma	Papadia et al. (2011)<br>Pantcheva et al. (2011)<br>Takagi et al. (2004)
**Timolol maleate 0.5%, 0.25% or timolol hemihydrate**	Non-selective β-adrenergic blocker<br>Less effective than brimonidine 0.2%<br>Reduces IOP by decreasing the secretion of aqueous humor not by increasing outflow facility<br>Downregulates the adenylate cyclase enzyme by inhibiting β2-adrenoceptor sites at the ciliary processes<br>Excreted in the urine, metabolized by CYP2D6<br>Used in combination	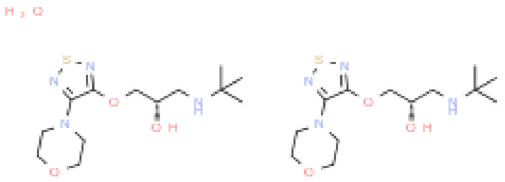	Severe adverse cardiovascular and respiratory effects<br>Dry eyes, local hypersensitivity reactions, blurred vision, induce conjunctival hyperemia, burning, stinging or superficial punctate keratitis, reduces tear flow<br>May cause bradycardia, arrhythmia, congestive heart failure, and syncope by Adam-Stokes syndrome<br>May induce anxiety, depression, sexual impotence, fatigue, confusion, disorientation, and hallucinations<br>Should be avoided in patients with lung disease, diabetic mellitus, or hypoglycemic attacks	Topical	Glaucoma	Adkins et al. (1998)<br>Watanabe et al. (1983)<br>Volotinen et al. (2011)
**Travoprost**	Prostaglandin F2α analogue<br>Isopropyl ester prodrug<br>Lowering IOP by increasing the outflow of aqueous humor via TM and uveoscleral pathways	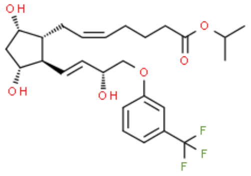	Eye irritation, dry eye, itching, blurred vision, burning, discharge, allergy, and blepharitis,<br>Iris pigmentation, hypertrichosis	Topical	Glaucoma	Arranz-Marquez et al. (2008)<br>Costagliola et al. (2009)
**Triamcinolone acetonide 25 mg**	Synthetic glucocorticoid	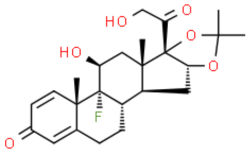	Risks of endophthalmitis, retinal tears/detachment, vitreous hemorrhage, elevated IOP, and cataract	Intravitreal	AMD<br>DR	Iu et al. (2007)<br>Vaziri et al. (2015)

**Trimetazidine**	Piperazine derivative<br>Selective inhibition the oxidation of free fatty acids and secondarily increases glucose oxidation<br>Enhancement of metabolic processes in the cell, counteraction of Na^{+} and Ca^{2+} accumulation and mitochondrial damage directly decreased ROS production<br>Antioxidative and anti-inflammatory effects: reduced the production of inflammatory cytokines in vitro via the regulation of the nuclear factor erythroid 2-related factor 2/heme oxygenase 1/caspase-8 pathway	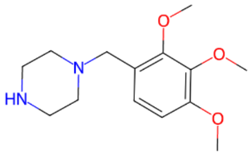	No known adverse effect	Oral	AMD<br>DR<br>Glaucoma	Pogatsa (2001)<br>Kaszuba-Barthowiak et al. (2008)<br>Novak et al. (2007)<br>Wan et al. (2017).
**Unoprostone isopropyl (Rescula®) 0.15%**	Prostaglandin analogue<br>Lowering IOP by increasing the outflow of aqueous humor via the uveoscleral pathway, but also it has some effect on trabecular outflow in animals	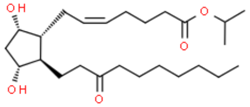	Conjunctival hyperemia (7%), corneal erosion (2%) and blepharitis (1%)<br>No effect on iris color	Topical	Glaucoma	Fung et al. (2014)

Color coding: Green: pharmacological agents take action on antiapoptotic and anti-aging mechanisms. Orange: pharmacological agents take action on anti-vessel formation mechanisms (anti-VEGF, anti-HIF1α). Yellow: pharmacological agents take action on anti-inflammatory mechanisms. Abbreviations: BBB = blood brain barrier, CNS = central nervous system, COX = cyclooxigenase, CRAO = central retinal artery occlusion, DR = diabetic retinopathy, ERG = electroretinography, FK506BP12 = FK506-binding protein-12, HDL = high density lipoprotein, IL = interleukin, IOP = intraocular pressure, MHC = major histocompatibility complex, mTOR = mammalian target of rapamycin, NF-κB = nuclear factor kappa B, NMDA = N-methyl-D-aspartate, RGC = retinal ganglion cell, RPE = retinal pigmentepithelium, TGF = transforming growth factor, TM = trabecular meshwork, VEGF = vascular endothelial growth factor
